# A phased genome assembly of a Colombian *Trypanosoma cruzi* TcI strain and the evolution of gene families

**DOI:** 10.1038/s41598-024-52449-x

**Published:** 2024-01-24

**Authors:** Maria Camila Hoyos Sanchez, Hader Sebastian Ospina Zapata, Brayhan Dario Suarez, Carlos Ospina, Hamilton Julian Barbosa, Julio Cesar Carranza Martinez, Gustavo Adolfo Vallejo, Daniel Urrea Montes, Jorge Duitama

**Affiliations:** 1 Systems and Computing Engineering Department, Universidad de los Andes, Bogotá, Colombia; 2 Laboratorio de Investigaciones en Parasitología Tropical (LIPT), Universidad del Tolima, Ibagué, Colombia; 3 School of Veterinary Medicine, Texas Tech University, Amarillo, TX 79106 USA

**Keywords:** Molecular evolution, Genome evolution, Evolutionary biology

## Abstract

Chagas is an endemic disease in tropical regions of Latin America, caused by the parasite *Trypanosoma cruzi*. High intraspecies variability and genome complexity have been challenges to assemble high quality genomes needed for studies in evolution, population genomics, diagnosis and drug development. Here we present a chromosome-level phased assembly of a TcI *T. cruzi* strain (Dm25). While 29 chromosomes show a large collinearity with the assembly of the Brazil A4 strain, three chromosomes show both large heterozygosity and large divergence, compared to previous assemblies of TcI *T. cruzi* strains. Nucleotide and protein evolution statistics indicate that *T. cruzi* Marinkellei separated before the diversification of *T. cruzi* in the known DTUs. Interchromosomal paralogs of dispersed gene families and histones appeared before but at the same time have a more strict purifying selection, compared to other repeat families. Previously unreported large tandem arrays of protein kinases and histones were identified in this assembly. Over one million variants obtained from Illumina reads aligned to the primary assembly clearly separate the main DTUs. We expect that this new assembly will be a valuable resource for further studies on evolution and functional genomics of *Trypanosomatids*.

## Introduction

Chagas disease (CD), also known as American trypanosomiasis, is a tropical disease caused by the protozoan parasite *Trypanosoma cruzi* that belongs to the Kinetoplastida class and the Trypanosomatidae family^1^. This disease is a public health problem, it is estimated that around 6–7 million people worldwide are infected with *T. cruzi*^2^. About 30,000 new cases are registered each year, an average of 12,000 deaths, and 9000 newborns are infected during pregnancy^3^. Particularly in Colombia, a prevalence between 700,000 and 1,200,000 infected people and 8,000,000 individuals at risk of acquiring the infection has been estimated^4^. A high prevalence has been reported mainly in the adult population, pregnant women^5^, and a high disease-related death rate has been reported in males^6^. Furthermore, in the period from 2012 to 2020, there was a fatality rate of 20% only in the Casanare region^7^.

CD is found mainly in endemic areas of 21 Latin American countries, including Colombia. However, in recent decades it has spread to other countries such as the United States, Canada, and some European and African countries, due to the migration of the infected population^2,8^ or the presence of the vector and parasite^9^.

The life cycle of the parasite is complex since it has several developmental stages in vectors and mammalian hosts. In the vector, it can be found as epimastigotes (replicative form) or metacyclic trypomastigotes (infective form). The metacyclic trypomastigotes enter the mammals through mucous membranes or breaks in the skin and transforms into amastigote (intracellular form) and then to the infective form to invade other cells^1,10^. More than 150 species of domestic or wild mammals, such as dogs, cats, rodents, common opossum and armadillos, can be reservoirs of the parasite^1,10^. In addition, about 152 species of triatomine insects are known to have the potential of acting as vectors of *T. cruzi*^11^.

*T. cruzi* is considered a parasite with a wide genetic diversity^12–14^. This parasite has been classified into 7 different Discrete Typing Units (DTU) TcI-TcVI and TcBat (genetically close to TcI)^15–19^. Additionally, subdivisions or genotypes within some DTUs such as *T. cruzi* I (TcI) have been suggested^20–25^, which demonstrates the wide genetic variability of the parasite. The different DTUs present associations with transmission cycles, geography, vector species, and clinical manifestation to a certain extent^19,26^. The variability of *T. cruzi* isolates circulating in Colombia and their association with the eco-epidemiology of Chagas disease have been studied for several years. The results show that the Colombian *T. cruzi* isolates present great genetic variability and suggest that TcI is predominant throughout the territory^27–30^. Particularly, TcI has been associated with heart disease in chagasic patients in Colombia^31^.

This extensive genetic variability is a result of the complexity of its genome. It has been widely reported that the genome of *T. cruzi* has extraordinary plasticity between strains, with a total length ranging between 40 and 140 Mb^32,33^. It is considered a generally diploid organism with the presence of aneuploidy in some hybrid strains^33–35^. Its proteome has about 22,000 proteins^36^. More than 50% of the genome consists of repetitive sequences, mainly represented by large multigene families encoding surface proteins, retrotransposons, telomeric repeats, and satellites^35,36^.

*T. cruzi* reference genomes have been highly fragmented and underrepresented for many years due to their genomic complexity and the nature of the data produced by short-read sequencing technologies^36–38^. For this reason, interest has grown in using long-read technologies to improve the assembly of repetitive regions. In recent years, some *T. cruzi* genomes have been published with these technologies (Supplementary Table 1), improving the understanding of the genome but have also shown the wide variability within and between DTUs and strains of the parasite^39–41^.

Although knowledge of the *T. cruzi* genome has been expanded in Colombia, there is no detailed description of the composition of the genome that includes the distribution of multigenic families and genetic diversity based on a genome sequenced with long reads technologies. Neither has the complete organization of the kDNA maxicircle been described in Colombian isolates belonging to the DTU TcI. In this paper, the sequencing of *T. cruzi* (TcI) isolated from Colombia was carried out using the new PacBio methodology—high fidelity (HiFi), and a description of the genome is reported that includes assembly, annotation, and also the genetic diversity between DTUs and comparative genomics.

## Results

### A phased genome assembly for the *T. cruzi* strain Dm25

We performed long read whole genome sequencing of epimastigotes of the *T. cruzi* strain Dm25 in exponential growth phase following the PacBio HiFi sequencing protocol. This platform provided 206,520 sequences with an average length of 20,997 bp. The median sequence quality was Q26, with all sequences having a quality greater than Q20. After evaluation of several options for genome assembly, we obtained partially phased assemblies from these reads running the tools Hifiasm and NGSEP. After evaluation and manual selection of the contigs, we built a combined phased assembly for Dm25.

The first haplotype (H1) can be considered a primary assembly and it is composed of 35 contigs representing 32 pseudo chromosomes and one copy of the maxicircle. Almost all chromosomes (30) were assembled in one single contig, and telomeric repeats [(TTAGGG)n] were identified on both chromosome ends for 24 single chromosome contigs and for the two chromosomes assembled in two contigs. The total length of this haplotype is 38.68 Mbp, and the median (N50) length is 1.23 Mbp (Supplementary Fig. 1). Performing quality assessment through mapping of conserved genes using BUSCO^42^, only one of the 130 conserved genes in Euglenozoa was fragmented. The remaining 129 genes were uniquely mapped to the assembly.

**Figure 1:**
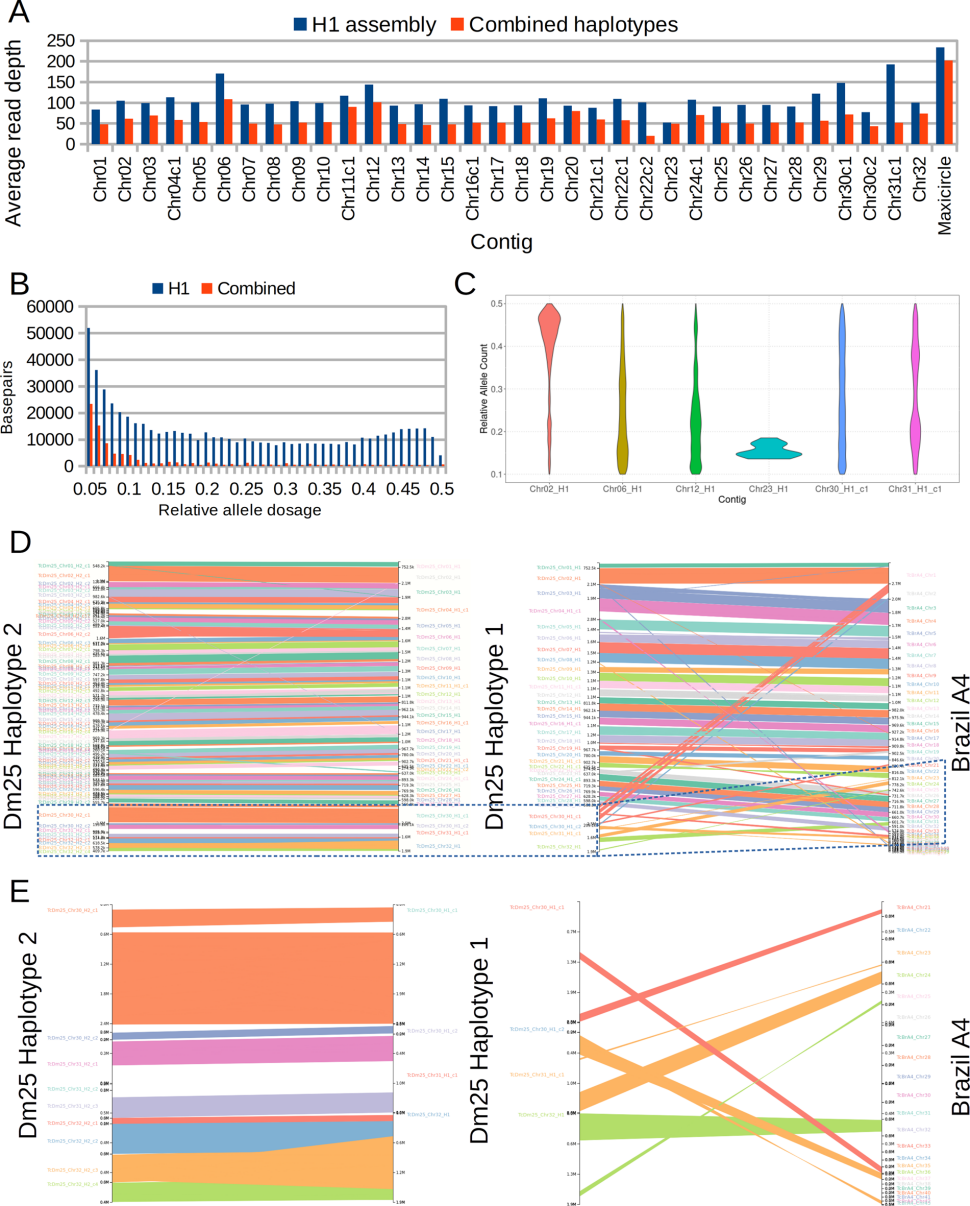
(**A**) Average read depth per contig of HiFi reads aligned to the primary haplotype and to the complete phased assembly. (**B**) Histogram of relative allele dosage in sites with two observed alleles after mapping to the haplotype H1 or to the combined assembly. (**C**) Violin plots of relative allele dosage in selected individual chromosomes. (**D**) Synteny-based alignment between the assembled haplotypes of the genome of Dm25 and between the haplotype H1 and the Brazil A4 genome assembly. (**E**) Zoom into the contigs assigned to chromosomes 30, 31, and 32 of Dm25.

To assess the ploidy of each contig, we realigned the reads to the H1 assembly and we calculated the average read depth for each chromosome, and the distribution of allele dosages for sites having more than one allele call. The read mapping rate to this assembly was 99.86%. Figure 1A shows that the average read depth varies around 100× for most contigs. Contigs assigned to chromosomes 6, 12, 30 and 31 are the main outliers with average read depths ranging from 144× to 192×, suggesting that chromosomes 6, 12 and 30 are triploid and chromosome 31 is tetraploid. Conversely, the average read depth of the contig assigned to chromosome 23 is about 51×, which suggests that only one copy of this chromosome is present in Dm25. Based on the average read depth, we predicted that between four and five copies of the maxicircle were present in Dm25.

Figure 1B shows the genome wide distribution of relative allele dosages in sites with more than one allele call. Although an increase in the number of sites can be observed for relative allele dosages over 0.4, which would suggest that the genome is diploid, the abundance of sites having intermediate relative allele dosages obscures this conclusion. Contig-specific distributions seem to explain this trend, supporting the aneuploidies predicted by the read depth distribution. Figure 1C shows these distributions for the contigs assigned to presumably aneuploid chromosomes 6, 12, 30, and 31 (see the distributions for all contigs in the supplementary Fig. 2). The figure also shows the distribution for a control diploid contig (chr02) and for the haploid contig assigned to chr23. The contig assigned to chromosome 6 has more sites with relative allele dosages between 0.3 and 0.4 than sites with dosages above 0.4, which is the expected behavior for chromosomes having three copies. However, it also has a large number of sites with relative allele dosages between 0.1 and 0.2, obscuring this prediction. The contig assigned to chromosome 12 has a decreasing distribution, which could correspond to a tetraploid chromosome in which sites with 3:1 relative allele dosage are much more frequent than sites with 1:1 dosages. The distributions of the contigs assigned to chromosomes 30 and 31 are complex to interpret, the former looking almost uniform over the range of allele dosages, and the latter having small peaks close to 0.2 and 0.4. The complexity of these distributions seem to be related with the abundance of repetitive elements within each chromosome and their status as core or disruptive contigs (details in the next section).

Considering the expected ploidy for each chromosome, we selected contigs from the automated assemblies to generate a second haplotype (H2). This haplotype was more fragmented having 96 contigs, which represents an average of about three contigs per chromosome (assuming that 32 is an accurate prediction of the base chromosome number). The total length in this case was 37.3 Mbp and the N50 was 572 kbp (Supplementary Fig. 1). Only five of the 130 conserved genes in *Euglenozoa* were not found in the second haplotype. Four of these genes are located in the haploid chromosome 23, which was included only in the first haplotype. The same gene fragmented in H1 was fragmented in this haplotype. Two copies were identified on the H2 reconstruction for two of the 124 remaining genes. The two duplications are interspersed and the genes correspond to the two Ubiquitin carboxyl-terminal hydrolases annotated in the *Euglenozoa* database. We also mapped the reads to this haplotype and calculated the average read depth (Supplementary table 2). Nine contigs had average depths below 75×, suggesting that some segments of the chromosomes 3, 15, 16 and 22 were not represented in H2. Conversely, 20 contigs had average depths above 125×, six of them assigned to chromosomes with predicted aneuploidies. Only four of the remaining contigs have lengths above 200 kbp. These are located in chromosomes 10, 11, and 19. The increased depth suggests that some repetitive structures located in these chromosomes could not be resolved with the same resolution achieved in the H1 contigs. Taking into account the possible aneuploidies and copy number variation suggested by the read depth analysis, we further generated a partial third haplotype (H3) composed of 83 small contigs adding to 8.05 Mbp. Most sequences (53 sequences adding to 5.32 Mbp) in this haplotype were assigned to the repetitive or possibly aneuploid chromosomes 4, 6, 12, 30 and 31. The final genome is a concatenation of these three haplotype assemblies and aims to represent the complete haplotype diversity within the Dm25 genome.

Searching for kinetoplastid molecules in the genome assembly of Dm25, we obtained a complete reconstruction of the maxicircle. After circularization and sorting, the total length of this assembly was 47,166 bp (Supplementary Fig. 3). We identified the 18 expected protein coding genes, which make up the entire conserved region of the maxicircle^43^. The total length of the conserved region is 15,429 bp (32.7% of the total) and the GC-content is 25.34%. Most of the region has an excess of cytosine relative to guanine in the positive strand, which is measured by a negative GC-skew statistic^44^. The remaining 31,737 bp (67.3% of the assembly) correspond to the variable region. The GC-content of this region (21.39%) is lower than that of the conserved region. Following previous works^45,46^, we divided this region into a small subregion (3660 bp), termed P5, and a long subregion (28,077 bp), termed P12. The two regions are composed of a series of tandem repeats with unit lengths around 150 bp for the short region, and 1500 bp for the long region. Nucleotide identity between pairs of repeat copies ranged from 75.6 to 100%. We identified 18 palindromic conserved elements in the P12 region, which are known to be involved in different molecular processes such as the replication of the mitochondrial DNA^46^.

We compared the assembled haplotypes of Dm25 with the genome assembly of the Brazil A4 isolate^41^ which is the current most accurate haploid assembly of a TcI strain. We did not use this genome to merge contigs to avoid misassemblies produced by true structural variation between Dm25 and A4 or by possible misassembly errors in Brazil A4. However, we used the Brazil A4 genome as a reference to sort and orient most contigs of the first haplotype that were clearly collinear with Brazil A4 contigs. We identified two types of contigs based on this comparison (Fig. 1D). One to one relationships could be identified between contigs of Dm25 and contigs of Brazil A4 for 30 of the 34 contigs of Dm25 H1. The remaining four contigs showed a high level of structural variation between the Dm25 and the Brazil A4 genome (Fig. 1E). The contigs assigned to chromosome 30 of Dm25 could be mapped to the contig termed chromosome 2 of Brazil A4. However, at least two different syntenies could be identified, suggesting a translocation of this chromosome between the strains, or misassembly errors, either in Dm25 or in Brazil A4. Moreover, the largest contig of Dm25 assigned to chromosome 30 included two complete copies of the contigs reported as chromosome 21 and chromosome 36 in the Brazil A4 genome. Consistent with Wang et al. (2021), we found that this chromosome is almost entirely composed of copies of the most repetitive gene families in *T. cruzi* (details in the next section)^41^. The contig assigned to chromosome 31 of Dm25, could be mapped partially to the contig termed chromosome 24 of Brazil A4. However, it also included complete copies of the contigs labeled as chromosome 37 and chromosome 43 of Brazil A4. The two chromosome copies of Dm25 could be assembled in one and three contigs, respectively. However, the synteny between the two copies was interrupted in the central part of the chromosome. A dotplot of the H1 contig and the combined contigs of H2 revealed that the region is composed of a complex repetitive structure (Supplementary Fig. 4). Finally, chromosome 32 of Dm25 (haplotypes assembled in one and four contigs) included segments syntenic with sequences termed as chromosomes 25, 32, 35, 38 and 39 of Brazil A4.

### Gene annotation and protein evolution in *Trypanosoma* species

We identified 47,772 TEs in the combined assembly, covering 36.4 Mbp (44%) of the assembly (Supplementary Table 3). The percentage of the genome covered by TEs was larger for the haplotype H1 (48%), compared to H2 (38%) and H3 (35%), probably because a larger number of copies of TEs could be assembled in the contigs included in H1, and hence H1 is more complete than H2. Most TEs (25,355 covering 24.3 Mbp of the genome) were annotated as “Unknown”.

**Figure 2:**
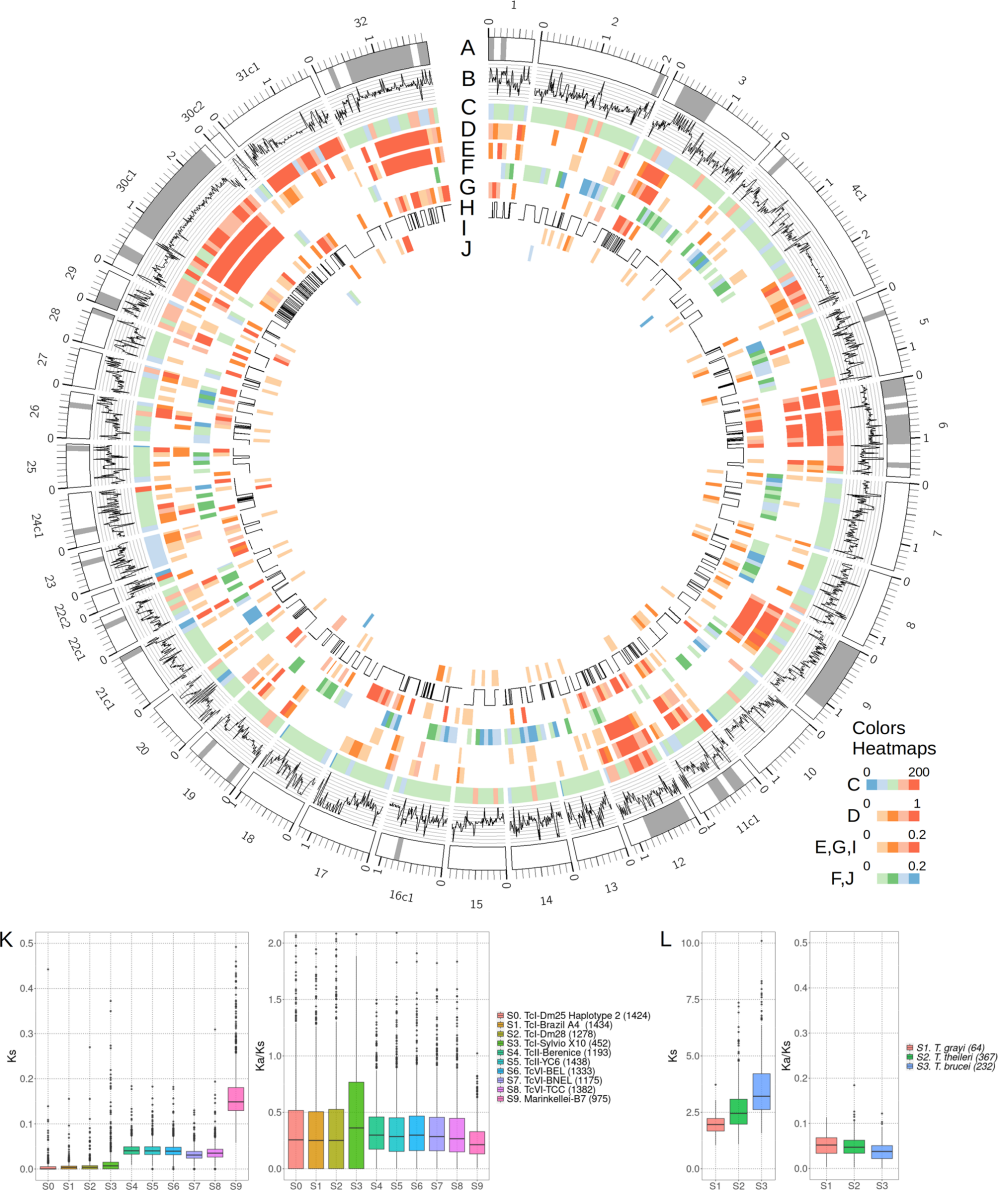
Distribution of genes and repeat elements in the primary assembly of the Dm25 strain. (**A**) Karyotype representation. Contigs with suffixes c1 or c2 have telomere repeats on only one side. Grey bands represent the disruptive compartment^39^. (**B**) GC-content over 10 kbp windows. Limits: 40–60%. (**C**) Average read depth in windows of 100 kbp. (**D**) Density of transposable elements. (**E–G**) Density of (**E**) gene families making the disruptive compartment (Transialidases, MASP and Mucin), (**F**) single copy genes. (**G**) Other known repeat families (DGF, GP63, RHS and TED). (**H**) Gene orientation, zero for negative and one for positive. Lines represent strand switches. (**I)** Density of protein kinases and (**J**) Density of histones. (**K**,**L**) Distributions of synonymous nucleotide divergence rate (ks) and relative non-synonymous to synonymous divergence rate (ka/ks) for pairs of orthologs, comparing the primary assembly of Dm25 (H1) with (**K**) other *T. cruzi* assemblies and (**L**) with assemblies of other species.

Following the pipeline implemented in the Companion website^47^, we annotated 29,544 genes for the complete assembly. The completeness of H1 translated into a larger number of annotated genes (14,207), compared to H2 (13,679). Synteny analysis between unique genes in these catalogs revealed 6762 genes with a clear syntenic copy on each haplotype. The gene catalog was complemented with 1658 additional genes annotated in H3. A total of 6659 genes (22.5%) were annotated as pseudogenes. Figure 2 summarizes the annotations in the main assembly (the H1 haplotype), including GC-content, strand switches, density of transposable elements, gene families and density of single copy genes. Consistent with previous analysis^39^ the genome can be distributed in regions that can be cataloged as “core”, enriched in single copy genes (Fig. 2F), and “disruptive” regions enriched in transposable elements and multicopy gene families (Fig. 2A,D,E). Whereas core regions have GC-contents around 0.5, disruptive regions seem to have a higher GC content, probably determined by the repetitive elements present in the regions (Fig. 2B). Strand switches also seem more frequent in disruptive regions, compared to core regions (Fig. 2H).

Compared to the available annotation of the Brazil A4 strain, the annotation of each haplotype has about the same number of genes. However, the lengths of the genes annotated in H1 and H2 (about 1.4 kbp) are on average about 200 bp larger than the lengths of the genes annotated in A4 (Supplementary Fig. 5). Consequently, proteins annotated in the two haplotypes are about 45 amino acids longer than those annotated in A4. We investigated the alignments of some of the single copy synteny homologs (or gene alleles) having the largest length differences and we found that most cases are produced by differences in coding start site or early stops, probably produced by point mutations or sequencing errors. The supplementary file 1 details the alignments of the three cases with longest differences on both sides of the distribution. The longest difference in favor of Dm25 is a hypothetical protein that includes a large tandem repeat structure. The structure was recovered independently in the contigs assigned to chromosome 5 in H1 and H2. The other two cases are located in chromosome 8 and 28 respectively and seem to be produced by early stop codons in the Brazil A4 homologs. The latter case seems to occur because the alleles in Dm25 are annotated with two separate CDS. Regarding genes with alleles longer in Brazil A4, the most extreme case is an endosomal trafficking protein located in chromosome 15. The difference seems to be produced by a mutation causing an early stop codon. Interestingly, the length difference is only observed in the H1 allele, whereas the H2 allele has a length similar to the Brazil A4 allele. The other two cases are a protein kinase and a hypothetical protein located in chromosomes 2 and 3, respectively. In both cases the difference seems to be produced by differences in the transcription start site. In the latter case, the difference is only observed in the H1 allele, whereas the H2 allele has a length similar to the Brazil A4 allele, only having an internal deletion of 15 amino acids.

Combining protein family annotations with homologies between Dm25 and annotated genes in the Brazil A4 transcriptome, we found a large and consistent number of copies of the main protein families: Transialidase (TS), Mucin-Associated Surface Protein (MASP), Retrotransposon Hot Spot (RHS), Mucin, Dispersed Gene Family (DGF) and Surface Protease GP63 (Supplementary Fig. 5C). Figure 2E,G show the distribution of these gene families across the genome, grouping families that make the disruptive genome (TS, MASP and Mucin) and other dispersed families (RHS, DGF and GP63), according to the classification proposed by Berna and collaborators^39^. Based on protein domains and orthology, we also identified large families of protein kinases (PKS, Fig. 2I) and core histone proteins (HIS, Fig. 2J). We observed a large overlap between the TS, MASP, RHS, DGF, and MUC families with annotated TEs. In contrast, the protein kinases did not overlap with annotations of transposable elements.

We analyzed the nucleotide and protein evolution between genes annotated in our assembly and genes annotated in other *T. cruzi* assemblies. Figure 2K shows the normalized differences in synonymous sites (Ks) comparing synteny orthologs of the haplotype H1 with other *T. cruzi* assemblies, including the haplotype H2. The Ks values are on average below 0.02 for comparisons with TcI assemblies, although a larger variance is observed in the comparison with the strain Sylvio, compared to H2, A4 and Dm28c. The Ks values obtained from orthologs with assemblies of other DTUs are significantly higher than those obtained from comparisons within TcI (p value < 10^–16^ of a Wilcoxon rank test). However, the absolute values are on average lower than 0.05, except for the comparison against the assembly of a *T. cruzi* marinkellei strain, for which the average increases to 0.15. In contrast, the Ks values of synteny orthologs between the first haplotype of Dm25 and the genes annotated in other species are on average above 2 (Fig. 2L). This suggests high divergence times between *T. cruzi* and other species with contiguous assemblies (*T. grayi*, *T. thelleri* and *T. brucei*).

We also investigated the behavior of protein evolution for single copy synteny orthologs within and between species. For each pair of unique synteny orthologs, we calculated the ratio between normalized non-synonymous and synonymous mutations (Ka/Ks). According to the neutral theory of evolution, this value should be close to 1 for genes not affected by selection. Values lower than 1 indicate purifying selection and values higher than 1 indicate positive selection and rapid protein evolution. Most Ka/Ks values for orthologs within species fall below 1 with averages between 0.3 and 0.5 (Fig. 2K), which suggests that most core genes are subject to some level of protein conservation through purifying selection. Theoretically, outliers that have Ka/Ks > 1 should correspond to genes with rapid protein evolution. However, close inspection of these outliers revealed that these cases were produced either by a very low ks proportion (zero in most cases) or by an over estimation of ka proportions produced by alignments around probably erroneous single base pair deletions. Conversely, the Ka/Ks values for orthologs between species were on average lower than 0.1 (Fig. 2L). This result which at first sight looks surprising can be explained because very few orthologs could be identified between these species, and hence these genes are probably those with the highest selective pressure for protein conservation.

**Figure 3:**
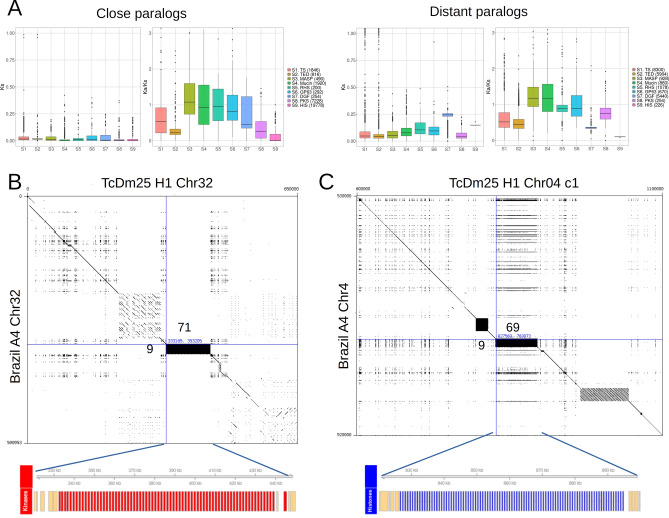
(**A**) Distributions of synonymous nucleotide divergence rate (ks) and relative non-synonymous to synonymous divergence rate (ka/ks) for pairs of paralogs of the six main multicopy gene families, and for protein kinases (PKS) and histones (HIS), discriminating tandem (close) paralogs from distant paralogs. (**B**) Alignment of the contig assigned to chromosome 32 of the haplotype H1 of Dm25 with the contig assigned to chromosome 32 of Brazil A4. The lower track highlights a tandem array of protein kinases spanning the black rectangle. The numbers besides the alignment indicate the number of copies annotated in the regions. (**C**) Alignment of the contig assigned to chromosome 4 of the haplotype H1 of Dm25 with the contig assigned to chromosome 4 of Brazil A4. The lower track highlights a tandem array of histones spanning the black rectangle. The numbers besides the alignment indicate the number of copies annotated in the regions.

Regarding multicopy gene families, Fig. 3A shows the distribution of Ks and Ka/Ks values, differentiating paralogs by physical proximity in the genome. The Ks values were significantly lower for close paralogs, compared to distant paralogs, suggesting that tandem duplications are more recent than interspersed duplications. The difference was larger for DGF paralogs, compared to other previously described families. This suggests that the diversification and spread of the DGF paralogs across the genome happened at a much older time compared to other families. Ka/Ks values were closer to 1 compared to the values obtained from single copy orthologs, suggesting that these genes are subject to a more relaxed purifying selection, compared to single copy genes. The averages for both close and distant paralogs of the MASP and Mucin families were larger than 1, suggesting a faster protein evolution, compared to the other families. Conversely, close paralogs of the TED, PKS and HIS families and distant paralogs of the DGF and HIS families have a Ka/Ks distribution similar to that observed for single copy orthologs. The latter case suggests that purifying selection is acting to preserve the protein sequence of DGF and HIS paralogs.

In contrast to the gene families described in previous studies, close paralog pairs of protein kinases (7228 pairs) were much abundant than distant paralog pairs (254 pairs). The reason for this is that most genes in this family resulted from a recent tandem duplication generating 71 copies of protein kinases spanning 200 kbp of chromosome 32 (Fig. 3B). The syntenic region in the haplotype H2 contains 67 homolog copies (Supplementary Fig. 6). This duplication event was not clearly observed in previous genome assemblies. A tandem array of 9 homologs in the Brazil A4 assembly are located in the syntenic region of the contig termed Chr32 (Fig. 3B). Besides this, 20 additional homologs are located in three contigs smaller than 32 kbp and not assigned to chromosomes. This suggests that a similar expansion could be present in the Brazil A4 assembly but that it could not be completely reconstructed due to technical limitations of the sequencing technology or the assembly pipeline. The comparison with the genome assembly of the Dm28c strain revealed a similar situation (Supplementary Table 4). Tandem arrays of 28 and 8 copies were identified in two contigs of 101 kbp and 22 kbp, respectively. Conversely, no orthologs were identified in the Sylvio genome assembly. Comparing with assemblies of strains belonging to other DTUs, tandem arrays of 12, 22, and 16 homologs were observed in one contig of the Berenice assembly and two contigs of the TCC assembly.

The annotation also revealed two large recent tandem duplications of core histones, located in chromosomes Chr04 (69 copies in H1, 33 copies in H2, Supplementary Fig. 7) and Chr18 (46 copies in H1, 43 copies in H2). This expansion is not observed in the Brazil A4 assembly (Fig. 3C). The assembly of the Sylvio strain was the only TcI assembly in which homolog tandem arrays were identified, having 95 copies homolog to the array on Chr04 and 34 copies homolog to the array in Chr18 (Supplementary Table 4). Two tandem arrays of 21 and 44 genes, both homologous to the array located on Chr18 were identified in two separate contigs of Dm28c. Regarding other DTUs, only the TCC assembly has three tandem arrays of 42, 19 and 22 genes. While the first array is homologous to the array on Chr04 of Dm25, the other two arrays are homologous to the array on Chr18. A closer look to the contig of the TCC assembly homologous to the region of chr04 (PRFC01000019) revealed that this contig seems to have evidence of the tandem array on chr04 (Supplementary Fig. 7). However, the homologous region is poorly covered by the publicly available gene annotation, and hence only three histones are annotated through this region.

### Genetic variability in *T. cruzi*

To evaluate the use of our haploid genome assembly as a reference for diversity studies, we reanalyzed publicly available WGS Illumina reads sequenced from 39 T*. cruzi* strains classified in different DTUs (Supplementary Table 5). The number of reads of the downloaded sequences was highly variable and the mapping rate ranged from 34% (2 samples) to 86% (Supplementary Fig. 8).

**Figure 4:**
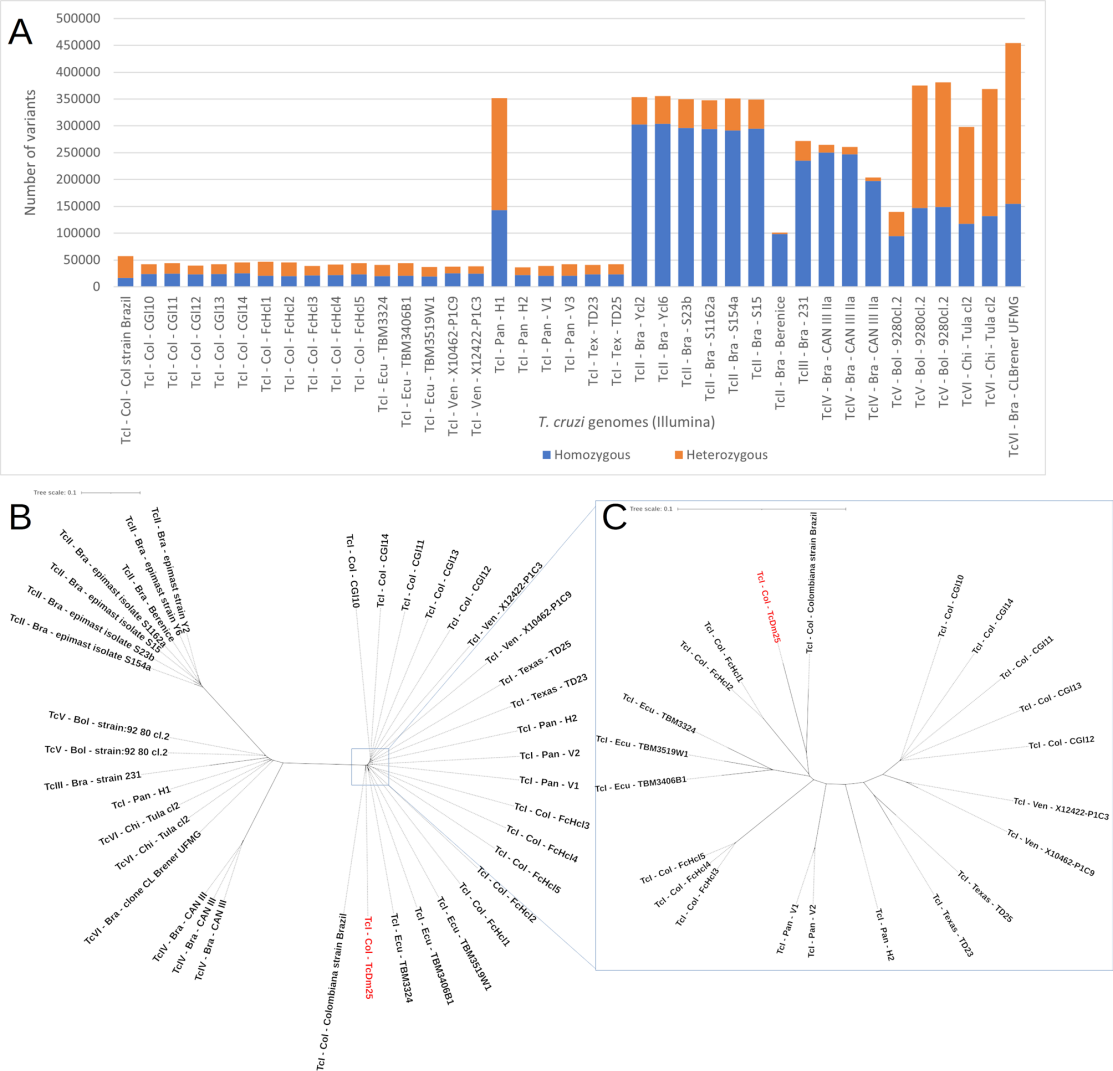
Genomic variants identified between Illumina reads of *T. cruzi* strains from different DTUs and our haploid *T. cruzi* assembly (H1). (**A**) Number of homozygous and heterozygous variants in *T. cruzi* strains*.* (**B**) Neighbor joining clustering of genetic distances between *T. cruzi* strains from different DTUs, including the haploid Dm25 assembly. (**C**) close up of the variability within TcI. The names indicate the DTU—country of isolation—strain. *Bra* Brazil, *Bol* Bolivia, *Pan* Panama, *Chi* Chile, *Col* Colombia, *Ven* Venezuela, *Ecu* Ecuador.

A raw dataset of 1,018,520 single nucleotide variants was found in all Illumina sequences mapped to the first haplotype of the Dm25 assembly. Figure 4A shows that the TcI group has the least number of variants, except for the H1 strain from Panama, which has about 6 times more variants. The number of variants of the H1 strain is similar to the variants of the TcV group. Strains Berenice and 9280cl.2 had a significantly lower number of variants compared to their groups, due to the low number of reads sequenced in these strains (Supplementary Fig. 8). In general, these results are consistent with previous molecular characterization showing that the Dm25 strain belongs to the TcI group.

We derived a neighbor joining clustering from the variants identified in the *T. cruzi* strains with Illumina reads (Fig. 4B). All the strains from TcI were grouped in the same node, except for the H1 strain from Panama, which is consistent with Fig. 4A and suggests that this strain is not TcI. Likewise, the TcII strains were grouped on the left side of the clustering. The TcIII, TcV, and TcVI groups were grouped in a node, together with the TcI strain from Panama. This node looks intermediate between TcI and TcII groups. Although the reads of Dm25 were not obtained by Illumina technology, we were able to confirm that Dm25 strain belongs to the TcI group with this analysis.

Figure 4C shows a close look at the clustering within the TcI group. The CGl strains, which are parasites isolated from a patient with HIV and cardiomyopathy, are clustered together on the right side of the figure. The FcHcI strains, which correspond to parasites isolated from an acute chagasic patient infected by oral transmission, formed two distinct groups. The Colombiana strain was placed close to Dm25 but the separation suggests a level of divergence between these strains larger than that observed within the other groups. In general, a grouping by countries is observed, except for the strains from Colombia.

## Discussion

The development of sequencing methods to obtain high-quality long read DNA sequencing data enabled the complete characterization of complex genomes, including phase reconstructions for diploid and even polyploid species. In this work, we report the nearly complete and phased assembly of a Colombian TcI strain of *T. cruzi*. The use of the PacBio HiFi technology allowed us to annotate and analyze a complete catalog of the most important repetitive structures, including transposable elements and multicopy gene families. Compared to previous efforts using the Oxford Nanopore and conventional PacBio technology^32,41,48^, the small error rate of HiFi reads allowed us to identify heterozygous sites, make inferences about ploidy per chromosome, and reconstruct two haplotypes for diploid and aneuploid chromosomes. A wide variability between the haplotypes of TcI and even within the TcI haplotypes was evidenced, especially for three chromosomes, two of them enriched for multicopy gene families and cataloged as part of the disruptive compartment. This confirms the broad plasticity of the genome and the strain-specific evolution of *T. cruzi* previously reported^32,39–41,48^. Further improvements in read quality and length of long read sequencing technologies will facilitate the full reconstruction of large numbers of strain genomes for pathogens with complex genomes such as those in the *Trypanosomatidae* family. This will allow researchers to characterize and analyze the function and evolution of the expectedly large haplotype variability segregating within *T. cruzi*.

The total length of the genome assembly of Dm25 (84 Mbp) is consistent with previous flow cytometry experiments in which the total genome size was estimated to fluctuate between 80 and 150 Mbp^49^. The percentage of the genome covered by repetitive elements (~ 47%) is also consistent with previous reports^35,41^, and only differs from the percentage reported for the Sylvio X10 strain, which was only 18.43%^48^. Even using long reads, heterozygosis and a large percentage of repetitive elements are the main difficulties to obtain chromosome-level genome assemblies^50^. Part of the complexity of the *T. cruzi* genome is evidenced by the presence of different aneuploidies. Recent studies also support the presence of aneuploidies in the genomes of TcI strains^51^. In particular, chromosome 31 seems to have a consistent increase in number of copies, compared to diploid chromosomes^35^. One of the characteristics of this chromosome is the abundance of genes related to protein glycosylation, such as mucin surface proteins. These proteins can be related to the survival of *T. cruzi* during the infection process^52,53^. Previous studies also have investigated the role of aneuploidies to facilitate a rapid adaptation of the pathogen across its life cycle, while moving from an invertebrate to a vertebrate host, through modulation of allele dosages^54,55^.

The number of gene copies per family identified within each haplotype was consistent with previous studies^39,41^. The analysis of nucleotide and protein evolution over paralog pairs provides insights into the relative times of expansion of the different families, and their level of protein conservation. With the exception of Mucins and dispersed gene families (DGF), the ks value in most comparisons between paralogs was below 0.2. The similarity of these distributions with the distribution of ks values for core orthologs against the *T. cruzi* marinkellei assembly, suggests that most of the observable expansions of gene families occurred along the diversification of the species. As expected, tandem paralogs seem to have appeared at a smaller time, compared to distant paralogs. The distribution of ka/ks values suggests that protein sequences of multicopy gene families evolve faster than core genes, thanks to a more relaxed purifying selection. A notable exception to this pattern is the case of distant copies of dispersed gene families (DGF). The expansion of this family seems to occur at a much older time, compared to the other families, but at the same time, the ka/ks values suggest a high level of protein conservation. High protein conservation and lack of positive selection in this family were previously reported in a study in which Shannon entropy was used as a measure of variability across a protein sequence alignment^56^. This is surprising taking into account that subtelomeric regions contain copies of DGF genes^57^. These regions usually have higher levels of homologous recombination and are even subject to ectopic recombination, which increases the variability of genes present in these regions^58^. Moreover, recent studies reported that many subtelomeric coding sequences of DGF genes serve as replication origins^59^, and that up to 80% of DGF genes include dynamic nucleosomes^60^. It has also been shown that DGF genes are expressed in the three life cycle stages^56,61^. We actually observed a significant enrichment of DGF genes in subtelomeric regions of the H1 assembly (32 out of 159, p value = 5.02 × 10^–7^ of a Fisher exact test). Further studies are needed to elucidate why the pathogen requires protein conservation for this family.

Beyond the well characterized repeat families, we observed three recent expansions of protein kinases and histones. A comparative analysis of public assemblies within the syntenic regions that could be identified suggests that these expansions are not a unique feature of Dm25, but that the expansions could not be fully characterized due to the lack of completeness of previous assemblies. Gene copies of the expansion of protein kinases in chromosome 32 belong to the TcCK1.2 gene family. This is a casein kinase 1 (CK1) which is a signaling serine/threonine protein. These proteins are involved in different cellular processes such as protein trafficking, cell cycle regulation, cytokinesis, DNA repair and apoptosis^62,63^. TcCK1.2 is more expressed in the amastigote stage, compared to the epimastigote and the trypomastigote stages^63^. Orthologs TcCK1.2 in *L. donovani, L. amazonensis*^64^ and *T. brucei*^65^ are crucial for parasite survival in vertebrate stages. This suggests that the expansion of TcCK1.2 could be related to an adaptation mechanism. Likewise, gene copies of the expansion of histones found in chromosome 4 belong to the histone variant H2B.V. Histones play a key role in the organization of the chromatin structure and gene expression in *T. cruzi*^66^. Previous studies analyzing chromatin extracts^67^, ChIP-seq data and performing knockout experiments^68^ showed that this variant is associated with nucleosome instability and that it is more expressed in epimastigotes compared to trypomastigotes. This suggests that H2B.V genes can be related to chromatin structure changes and modulation of transcription rates^69^. Further functional experiments are needed to reveal the relationship between gene copy number and expression changes during host–pathogen interactions.

Nucleotide evolution statistics on core orthologs suggest that there is a large gap in the range of species that need to be sequenced to obtain a full reconstruction of the evolutionary history of *Trypanosomatidae*. The closest species to *T. cruzi* that we could identify with a publicly available genome was *T. grayi*. The average ks values for core ortholog pairs were close to two, suggesting a very large divergence time between these species. High protein conservation was observed in these paralogs, suggesting that only ultraconserved essential proteins were included in this comparison.

The use of high-quality long reads allowed to obtain a complete and direct assembly of the maxicircle, without any scaffolding or curation steps. The complex pattern of long and short repetitive elements present in the divergent region explains why the Maxicircle can not be fully reconstructed using short read technologies^70,71^. The total length of our assembly (47 kbp) is within the range between 35 and 51 kbp estimated by previous studies^45^. The organization of the molecule in a gene rich conserved region and two divergent and repeat rich regions is also consistent with previous assemblies. Previous studies in *T. brucei* showed that this region contains binding sites for the topoisomerase II, which indicates that this region is important for the replication of the molecule^72^. Recent studies in *T. vivax* indicate that the variable region can have a large variability within species because copies of repetitive elements can recombine, producing presence/absence variants and even rearrangements^73^.

Based on the reanalysis of publicly available Illumina data, we show that the primary assembly of Dm25 can serve as a reference for population genomic studies in *T. cruzi*. A wide variation in the number of variants in the different DTUs was evidenced, which is consistent with the reported genomic heterogeneity^48,41^. Sample clustering based on SNP variation separates the reported DTUs^74^. A single NJ cluster includes the TcIII, TcV, and TcVI groups because TcV and TcVI are hybrids of TcIII and TcII^74^ although a more comprehensive sampling within DTUs is needed to corroborate this hypothesis. The genetic proximity between TcIII, TcV and TcVI strains has been reported in previous studies with markers such as gGAPDH where it has been impossible to separate hybrid strains from parental strains^75^. In addition, it was possible to corroborate the erroneous assignment of the H1 strain (from Panama) to the TcI group^76^. We expect that this resource will be very valuable for different research groups performing evolutionary, functional and population genomic analysis in *T. cruzi* and other related tropical pathogens.

## Methods

### Sampling area and parasite culture

The capture of the host *Didelphis marsupialis* was carried out in the municipality of Coyaima, department of Tolima (coordinates 3.8025-75.19833), using baited tomahawk traps (Supplementary Fig. 9). The collected specimen was sedated and individualized, to later take a blood sample, with the purpose of performing a blood culture in a biphasic medium (NNN: Novi, Nicolle, McNeal / LIT: Liver Infusion Tryptose). The specimen was liberated after checking that vital signals were stable. The research was conducted following all applicable guidelines and regulations for experiments involving animals. All experimental protocols followed in this research were approved by the bioethics committee for scientific research at Universidad del Tolima.

The strain of *T. cruzi* isolated was then cryopreserved in liquid nitrogen at the Laboratorio de Investigaciones de Parasitología Tropical (LIPT) of Universidad del Tolima until use. The isolate identified as Dm25 was thawed and placed in NNN culture medium with LIT supplemented with 15% fetal bovine serum (FBS) and 100 IU/ml gentamicin/ampicillin mixture at 28 °C for its log-phase growth, allowed obtaining 10^8^ parasites for the molecular characterization and whole genome sequencing (WGS).

### Molecular characterization

Species identification was based on the amplification of the hypervariable region of trypanosomatid minicircles using primers S35 (5-AAA TAA TGT ACG GGT GGA GAT GCA TGA-3), S36 (5-GGG TTC GAT TGG GGT TGG TGT-3) and KP1L (5-ATA CAA CAC TCT CTA TAT CAG G-3) as proposed by Vallejo et al.^77^. The amplification products were visualized by electrophoresis with 6% polyacrylamide gels, stained with silver nitrate, and 1 kb Plus DNA Ladder (Invitrogen ™ by ThermoFisher Scientific, Product 10787018). The protocol proposed by Brisse and collaborators^78^ was followed for the genotyping of the *T. cruzi* isolate within a Discrete Taxonomic Unit (DTU). This protocol starts amplifying the intergenic region of the spliced-leader gene (SL-IR) using the primers proposed by Souto et al.^79^, which amplifies a product of 300 bp corresponding to DTU II and 350 bp corresponding to DTU I. Because Dm25 was cataloged within the DTU I lineage by this initial test, no further testing was applied with other primers. The amplification products were visualized in agarose gel electrophoresis stained with 2% Ethidium Bromide.

### DNA extraction and genome sequencing

DNA extraction from *T. cruzi* epimastigotes was performed using the Gentra Puregene kit (Qiagen) to obtain high molecular weight DNA. The DNA was quantified with the NanoDrop 2000 spectrophotometer (Thermo Scientific, USA). The integrity of the DNA was verified by electrophoresis with a 2% agarose gel (90 V for 30 min). *T. cruzi* DNA sequencing was performed using Pacific Bioscience (PacBio) HiFi technology at 100× average read depth.

### Sequence assembly and quality assessment

Raw phased de novo assemblies of the *T. cruzi* strain Dm25 were obtained running two different software tools for phased genome assembly. Hifiasm v0.12(r304)^80^ was executed with the parameter “-n-hap” equal to 2 to assemble a diploid genome. The Assembler command of the Next Generation Sequencing Experience Platform (NGSEP) v4.3.1 was also executed using as parameters a k-mer length (-k) of 25, window length (-w) 40 and ploidy (-ploidy) of 2^81^. Contigs were aligned to the publicly available haploid genome assembly of the TcI Brazil A4 strain using Minimap2 v2.22^82^. Based on these alignments, the contigs of both haplotypes were manually sorted and oriented using a custom script available with the NGSEP distribution (class ngsep.sequences.io.FastaSortAndRenameSequences). Minimap2 alignments were also performed within and between raw genome assemblies, and raw reads were also aligned to the two assemblies using minimap2. Heterozygous variants were called from aligned reads using the SingleSampleVariantsDetector command of NGSEP, to assess the capacity of each assembly to separate the reads sequenced from the two haplotypes of each chromosome. For each chromosome, contigs making the haplotypes H1 and H2 were selected manually based on assembly length, presence of telomeric repeats, genetic distance between haplotypes (as predicted by minimap2), agreement between assemblies on possible structural variants against the A4 assembly, and amount of heterozygous variants.

To identify contigs belonging to a possible third haplotype in aneuploid chromosomes or contigs related to unresolved repeats, we aligned the PacBio reads to the concatenated H1/H2 assembly using minimap2, called heterozygous variants using the SingleSampleVariantsDetector command of NGSEP, and calculated mean read depths on non overlapping 1 kbp regions using the depth command of samtools v1.16^83^. Density of heterozygous SNPs over 100 kbp regions was assessed using the VCFVariantDensityCalculator command of NGSEP. Windows with presence of heterozygous calls were manually verified visualizing the reads with the Integrative Genomics Viewer^84^ to discard regions with false positive calls produced by sequencing errors. Unassigned contigs were also mapped to the H1/H2 assembly to select contigs likely to belong to the third haplotype or to an unresolved repeat within the regions highlighted by the previous analysis. The complete process was repeated five times on concatenated genomes including the selected H3 contigs to validate that the H3 contigs will have good read coverage, that heterozygosity is reduced within the regions highlighted by the previous analysis and that the read depth on these regions reduces close to the global average of 50× per haplotype.

Genome statistics were obtained running QUAST v5.02^85^. Per base quality assessment through mapping of conserved genes was assessed using BUSCO v5.3.2 searching the reference dataset of 130 genes in Euglenozoa^42^.

### Genome annotation

We generated an initial database of Transposable elements using Repeat Modeler^86^. We mapped this initial database to the genome using Repeat Masker (http://www.repeatmasker.org/). We also executed the TransposonsFinder command of NGSEP^87^ to map the database generated with Repeat Modeler and annotate the regions including transposable elements in the genome assembly of *T. cruzi* (Dm25). We executed a separate process for each haplotype and executed two rounds of identification.

We ran the Companion software for structural and functional annotation of the genome of *T. cruzi*^47^*.* Companion combines the tool RATT^88^ to transfer models of publicly available assemblies, with ab-initio predictions obtained with SNAP^89^, and AUGUSTUS^90^. Functional annotation is performed by transferring annotations from orthologs obtained running OrthoMCL^91^, and performing blast searches to the Pfam-A database^92^. Additionally, this software takes into account that the genes of kinetoplastids such as *Trypanosoma* and *Leishmania* are organized in large directional groups of genes that are transcribed together as polycistrons. Hence, this software has a filtering method to eliminate the over-prediction of genes on the complementary strand. Genes belonging to multicopy gene families were identified combining genes with direct annotations of characteristic PFam domains with orthologs of genes in the Brazil A4 strain belonging to the family.

### Ploidy determination

Absolute chromosomal ploidy of *T. cruzi* assemblies was determined by estimating allele frequencies from the proportion of occurrence of each heterozygous site using the RelativeAlleleCounts command of NGSEP^93^.

### Genome alignments and sequence evolution statistics

Pairwise genome-wide comparisons between the haplotypes of Dm25 and among the publicly available genome assemblies included in this study were performed running the GenomesAligner command of NGSEP^94^ with kmer length (option -k) of 5 and weighted percentage of shared kmers (option -p) of 30. Assemblies were selected from the TritrypDB database of VEuPathDB^95^ based on their contiguity, which is related to the use of Sanger or long-read technologies. Dotplots of local alignment were performed using Gepard v1.40^96^. To calculate nucleotide and protein evolution statistics (Ks and ka/ks) the DNA coding sequences of homologs inferred from each pairwise comparison and having a length difference below 20% of the shortest sequence were aligned using a codon-aware pairwise aligner available with the NGSEP distribution (class ngsep.transcriptome.CodonCDSPairwiseAlignment). Alignments were provided to the command codeml of paml v4.9j^97^ for estimation of ka, ks and ka/ks proportions.

### Determination* of variants between DTUs of T. cruzi*

To determine variants between *T. cruzi* genomes from different DTUs, illumina reads sequenced from 33 T*. cruzi* isolates were downloaded from the TriTrypDB database (https://tritrypdb.org) (Supplementary Table 5)^76^. The mapping of Illumina reads to Dm25 H1 haplotype assembly was performed using the ReadsAligner command of NGSEP 4.3.1. Variants were discovered and genotyped using the MultisampleVariantsDetector command of NGSEP. Functional annotation, filtering and summary statistics of the variation database were performed using the commands VCFAnnotate, VCFFilter and VCFSummaryStats of NGSEP. Finally, a distance matrix was made from the variants using the VCFDistanceMatrixCalculator of NGSEP. In brief, this command calculates a identity-by-state distance between each pair of samples adding one for each heterozygous difference and two for each homozygous difference. Total differences are normalized by the total number of variants genotyped for each pair of samples. A neighbor joining tree was built using the command NeighborJoining of NGSEP and the tree was visualized in the iTOL tool^98^.

### Maxicircle genome assembly and annotation

We used BLAST+ (v2.11.0)^99^ to search known maxicircle sequences in the assembly obtained with NGSEP. Known maxicircles were downloaded from the databases NCBI nucleotide and TriTrypDB. We used EMBOSS^100^ to filter out contigs with GC-content less than expected. The maxicircle was manually annotated, looking for base pair level synteny between the assembly and the annotated maxicircle sequences using BLAST and ARTEMIS v. 18.1.0^101^. These tools were also useful to identify and deduplicate repeated extremes and to orient the contig. BLAST+ v2.11.0) was also run with a maximum e-value of 10^–6^ to find tandem repeats and define the variable regions. The annotated sequence was visualized using Circos v0.69^102^.

### Supplementary Information

Supplementary Information 1.

Supplementary Information 2.

Supplementary Information 3.

Supplementary Information 4.

Supplementary Tables.

## Data Availability

The data used in this study is available at the NCBI sequence read archive (SRA) database (https://www.ncbi.nlm.nih.gov/sra) with bioproject accession number PRJNA994590. The genome assembly is available at the Assembly database of NCBI (https://www.ncbi.nlm.nih.gov/assembly/) under the bioproject accession numbers PRJNA1039287 and PRJNA1039288. The genome and the companion annotation are also available as supplementary materials (Supplementary files 2, 3).
