# Supplementary material for: A phased genome assembly of a Colombian Trypanosoma cruzi TcI strain and the evolution of gene families
Source: Sci Rep. 2024 Jan 24;14:2054. doi: 10.1038/s41598-024-52449-x (PMC10808112; doi:10.1038/s41598-024-52449-x)
Supplement: Supplementary file 1 — Supplementary Information 1. [file 41598_2024_52449_MOESM1_ESM.pdf]

Case 1. hypothetical protein. Chr05. Span difference: 1296 bp.

```
TcBrA4_0102720-RA          MWSLIKDVCGTVPQAVAPRKERSNAGPRSHDERQQPLSSGLAGVGDFLSGWWDTINKKTK
TcDm25H1_000366000.1       MWSLIKDVCGTVPQAVAPRKERSNAGPRSHDERQQPLSSGLAGVGDFLSGWWDTINKKTK
TcDm25H2_000348700.1       MWSLIKDVCGTVPQAVAPRKERSNAGPRSHDERQQPLSSGLAGVGDFLSGWWDTINKKTK
                           ************************************************************

TcBrA4_0102720-RA          SFLRDTLGTSDIDAYDEPLPLLLRLTYEQREAMHVDEIIREISEAVSVIAIRSDRVHREA
TcDm25H1_000366000.1       SFLRNTLGTSDIDAYDEPLPLLLRLTYEQREAMHVDEIIREISEAVSVIAIRSDRVHREA
TcDm25H2_000348700.1       SFLRDTLGTSDIDAYDEPLPLLLRLTYEQREAMHVDEIIREISEAVSVIAIRSDRVHREA
                           ****:*******************************************************

TcBrA4_0102720-RA          RFLLSLSLEEQIAHTSDFINCYDWWERLVNRRLSSCQEILHNCQCPPESEESHTKCLLNQ
TcDm25H1_000366000.1       RFLLSLSLEEQIAHTSDFINCYDWWERLVNRRLSSCQEILHNCQCPPESEESHTKCLLNQ
TcDm25H2_000348700.1       RFLLSLSLEEQIAHTSDFINCYDWWERLVNRRLSSCQEILHNCQCPPESEESHTKCLLNQ
                           ************************************************************

TcBrA4_0102720-RA          RIYSIREKCTQDMATIASRGRILRDERYDILEKIPCGDQTKETAYMRHQIKKTGITEESI
TcDm25H1_000366000.1       RIYSIREKCTQDMATIASRGRILRDERYDILEKIPCGDQTKETAYMRHQIKKTDITEESI
TcDm25H2_000348700.1       RIYSIREKCTQDMATIASRGRILRDERYDILEKIPCGDQTKETAYMRHQIKKTDITEESI
                           *****************************************************.******

TcBrA4_0102720-RA          SRINQHDEFNKEDVLRQLTDKRIQKKGSDIRKTTNSKTNSTNTKEEANILVKEAEARRLA
TcDm25H1_000366000.1       SRINQHDEFNKEDVLRQLTDKRIQKKGSDIRKTTNSKTNSTNTKEEANILVKEAEARRLA
TcDm25H2_000348700.1       SRINQHDEFNKEDVLRQLTDKRIQKKGSDIRKTTNSKTNSTNTKEEANILVKEAEARRLA
                           ************************************************************

TcBrA4_0102720-RA          EEAAEARRLAEEAEARRLTEEAAEARRLTEEAEARRLAEEAEARRLTEEAEARRLAEEAE
TcDm25H1_000366000.1       EE-AEARRLAEEAEARRLTEEAAEARRLAEEAEARRLAEEAEARRLAEEAEARRLAEEAE
TcDm25H2_000348700.1       EE-AEARRLAEEAEARRLTEEAAEARRLAEEAEARRLAEEAEARRLAEEAEARRLAEEAE
                           ** *************************:*****************:*************

TcBrA4_0102720-RA          ARRLAEE-AEARRLAEEAEARRLAEEAEARRLAEE-AEARRLTEEAADVLLFCFLTAR-R
TcDm25H1_000366000.1       ARRLAEEAAEARRLAEEAEARRLDEEAEARRLAEEAAEARRLAEEAAEARRLAEEAAEAR
TcDm25H2_000348700.1       ARRLAEEAAEARRLAEEAEARRLDEEAEARRLAEEAAEARRLAEEAAEARRLAEEAAEAR
                           ******* *************** *********** ******:****:.  :.  :*  *

TcBrA4_0102720-RA          RASAFLSQATRFC-------FLSRRRASALPQQATCFCCFLSQATCFCCFLSQATCFCCF
TcDm25H1_000366000.1       RLAEEAAEARRLAEEAAEARRLAEEAAEARRLAEEAEARRLAEEAAEARRLTEEAAEARR
TcDm25H2_000348700.1       RLAEEAAEARRLAEEAAEARRLAEEAAEARRLAEEAEARRLAEEAAEARRLTEEAAEARR
                           * :   ::* *:.        *:   *.*      . .  *:: :. .  *:: :. .

TcBrA4_0102720-RA          LSQATCFCCFLSQ--ATCFCFLSQATCFCCFLSQATRFCCFLSQATCFCFLSQATCFCCF
TcDm25H1_000366000.1       LTEEAAEARRLAEEAAEARRLAEEAAEARRLTEEAAEARRLAEEAAEARRLAEEAAEARR
TcDm25H2_000348700.1       LTEEAAEARRLAEEAAEARRLAEEAAEARRLTEEAAEARRLAEEAAEARRLAEEAAEARR
                           *:: :. .  *::  * .  : .:*:    : .:*:    : .:*:    *:: :. .

TcBrA4_0102720-RA          LSQATCFC---FPQQGDVLLLSSAPGDVLLLLSQATCFCFLSQRLVLLLTATALLPQQAR
TcDm25H1_000366000.1       LAEEAAEARRLTEEAAEARRLAEEAAEARRLAEEAA----EARRLAEEAEARRLAEEAAE
TcDm25H2_000348700.1       LAEEAAEARRLTEEAAEARRLAEEAAEARRLAEEAA----EARRLAEEAEARRLAEEAAE
                           *:: :. .     : .:.  *:. ..:.  * .:*:     :.**.    *  *  : *

TcBrA4_0102720-RA          ASALPQSGDCFCFLSQATCFCCFLSQATCFCCFL----SQATRFCCFLSQATRFCCFLSQ
TcDm25H1_000366000.1       ARRLTEEAAEARRLTEEAAEARRLAEEAAEARRLAEEAAEARRLTEEAAEARRLAEEAAE
TcDm25H2_000348700.1       ARRLTEEAAEARRLTEEAAEARRLAEEAAEARRLAEEAAEARRLTEEAAEARRLAEEAAE
                           *  *.:..     *:: :. .  *    ::* *:    ::* *:.   ::

TcBrA4_0102720-RA          ATCFCCFLSQATRFCCFLSQATRFCFLSQATCFCCFLSQATCFCFLSQATCFCCFLSQAT
TcDm25H1_000366000.1       ARRLTEEAAEARRLAEEAAEARRLA--EEAAEARRLAEEAAEARRLTEEAAEARRLAEEA
TcDm25H2_000348700.1       ARRLTEEAAEARRLAEEAAEARRLA--EEAAEARRLAEEAAEARRLTEEAAEARRLAEEA
                           *  :    ::* *:.   ::* *:.  .:*:    : .:*:    *:: :. .  *:: :

TcBrA4_0102720-RA          RFCCFLSQATRFCCFLSQATCFCCFLSQATCFCFLSQATC----FCFLSQATCFCCFLSQ
TcDm25H1_000366000.1       AEARRLTEEAAEARRLAEEAAEARRLAEEAEARRLAEEAAEARRLAEEAEARRLAEEAAE
TcDm25H2_000348700.1       AEARRLTEEAAEARRLAEEAAEARRLAEEAEARRLAEEAAEARRLAEEAEARRLAEEAAE
```

```
                            .  *:: :  .  *:: :. .  *:: :    *:: :.     :.  ::*  :.    ::

TcBrA4_0102720-RA         ATRFCFLSQATCFC--------FLSQATCFCFLSQATCFCFL--------------SQAT
TcDm25H1_000366000.1      ARRLAEEAEARRLAEEAAEARRLAEEAEARRLAEEAAEARRLAEEAAEARRLAEEAAEAR
TcDm25H2_000348700.1      ARRLAEEAEARRLAEEAAEARRLAEEAEARRLAEEAAEARRLAEEAAEARRLAEEAAEAR
                          * *:.  ::*  :.        : .:* .  : .:*:    *             ::*

TcBrA4_0102720-RA         RFCFLSQATRFC--------FLSQATCFCFLSQATCFA------SSARRRFC--------
TcDm25H1_000366000.1      RLAEEAEARRLAEEAAEARRLAEEAAEARRLTEEAAEARRLAEEAAEARRLAEEAAEARR
TcDm25H2_000348700.1      RLAEEAEARRLAEEAAEARRLAEEAAEARRLTEEAAEARRLAEEAAEARRLAEEAAEARR
                          *:.  ::* *:.        : .:*:    *:: :. *      ::  **:.

TcBrA4_0102720-RA         FLSQATCFCLPQSAT-----CSAASSVRRRASAASSAR---------RRASAFLSQATCF
TcDm25H1_000366000.1      LAEEAEARRLAEEAAEARRLTEEAAEARRLTEEAAEARRLAEEAAEARRLAEEAAEARRL
TcDm25H2_000348700.1      LAEEAEARRLAEEAAEARRLTEEAAEARRLTEEAAEARRLAEEAAEARRLAEEAAEARRL
                          : .:* .  *.:.*:      . *:..** :. *:.**         ** :   ::*  :

TcBrA4_0102720-RA         CCFLSQATRFC--------FLSQATCFCFLSQATCFCCFLS----QATRFCSSSARRRAS
TcDm25H1_000366000.1      TEEAAEARRLAEEAAEARRLAEEAAEARRLAEEAAEARRLTEEAAEARRLAEEAAEARRL
TcDm25H2_000348700.1      TEEAAEARRLAEEAAEARRLAEEAAEARRLAEEAAEARRLTEEAAEARRLAEEAAEARRL
                              ::* *:.        : .:*:    *:: :. .  *:    :* *:...:*  *

TcBrA4_0102720-RA         AAPPARLRCCCCFLSQATCFCCFLSQATCFCCFLSQATRFCFLSQATCFCFLSQATCFCF
TcDm25H1_000366000.1      AEEAAEARRLAEEAAEARRLTEEAAEARRLAEEAAEARRLA-EEAAEARRLAEEAEARRL
TcDm25H2_000348700.1      AEEAAEARRLAEEAAEARRLTEEAAEARRLAEEAAEARRLA-EEAAEARRLAEEAEARRL
                          *  .*  *  .   ::*  :    ::*  :.   ::* *:.  . * .  : .:* .  :

TcBrA4_0102720-RA         LSQATRFCFLSQATCFCFSARRRASAASSARRRASASSVRRRAS--ASSARRRASAASSV
TcDm25H1_000366000.1      AEEAAEARRLTEEAA---EARRLTEEAAEARRLAEEAAEARRLAEEAAEARRLTEEAAEA
TcDm25H2_000348700.1      AEEAAEARRLTEEAA---EARRLTEEAAEARRLAEEAAEARRLAEEAAEARRLTEEAAEA
                           .:*:    *:: :.   .*** :. *:.*** *. ::  ** :  *:.*** :. *:..

TcBrA4_0102720-RA         RRRASAASSVRRRASAASSARRRASASSARRRASAAS-------QPGDALLLPQQATCFC
TcDm25H1_000366000.1      RRLAEEAAEARRLAEEAAEARRLAEEAEARRLTEEAAEARRLTEEAAEARRLAEEAAEAR
TcDm25H2_000348700.1      RRLAEEAAEARRLAEEAAEARRLAEEAEARRLTEEAAEARRLTEEAAEARRLAEEAAEAR
                          ** *. *:..** *. *:.*** *. :.*** :. *:       :..:*  *.::*:

TcBrA4_0102720-RA         FLSQATCFC---CSSAGDALLL--------------------------------------
TcDm25H1_000366000.1      RLAEEAAEARSLTEEAAEARRLAEEAAEARRLTEEAAEARRLAEEAAEARRLAEEAAEAR
TcDm25H2_000348700.1      RLAEEAAEARRLTEEAAEARRL--------------------------------------
                           *:: :. .    ..*.:*  *

TcBrA4_0102720-RA         ------------------------------------------------------------
TcDm25H1_000366000.1      RLTEEAAEARRLAEEAAEARRLTEEAAEARRLAEEAAEARRLTEEAAEARRLAEEAAEAR
TcDm25H2_000348700.1      ------------------------------------------------------------


TcBrA4_0102720-RA         ------------------------------------------------------------
TcDm25H1_000366000.1      RLAEEAAEARRLAEEAAEARRLTEEAAEARRLAEEAAEARRLAEEAAEARRLAEEAEARR
TcDm25H2_000348700.1      ------------------------------------------------------------


TcBrA4_0102720-RA         -------------------------PQSRRRASASSARRRASAFLSQATCFCFLSQATAL
TcDm25H1_000366000.1      LAEEAAEARRLTEEAAEARRLTEEAAEARRLAEEAAEARRLAEEAAEARSLT-EEAAEAR
TcDm25H2_000348700.1      ---------------------TEEAAEARRLAEEAAEARRLAEEAAEARSLT-EEAAEAR
                                                .::** *. ::  ** :   ::* .:    . * *

TcBrA4_0102720-RA         LLPQPGDALLLPQQATRFCFLSR-----RRLLLPQPGAR--ASASSARRRASASSARRRA
TcDm25H1_000366000.1      RLAEEAEARRLAEEAAEARRLAEEAAEARRLAEEAAEARRLAEEAEARRLAEEAAEARRL
TcDm25H2_000348700.1      RLAEEAEARRLAEEAAEARRLAEEAAEARRLAEEAAEARRLAEEAEARRLAEEAAEARRL
                           *.: .:*  *.::*:    *:      ***    . **  *. :.*** *. ::  **

TcBrA4_0102720-RA         S--ASSARRRASASSARRRAS--ASSARRRASAASSARRRASAASSVRRRASASSARRRA
TcDm25H1_000366000.1      AEEAAEARRLAEEAAEARRLAEEAAEARRLTEEAAEARRLAEEAAEARRLTEEAEARRLA
TcDm25H2_000348700.1      AEEAAEARRLAEEAAEARRLAEEAAEARRLTEEAAEARRLAEEAAEARRLTEEAEARRLA
```

```
                          :  *:.*** *. ::  ** :  *:.*** :. *:.*** *. *:..** :. :.*** *

TcBrA4_0102720-RA         SASSVRRRAS------------ASSARRRASASSARRRA-----------SASSARRRAS
TcDm25H1_000366000.1      EEAAEARRLAEEAAEARRLAEEAAEARRLAEEAEARRLAEEAAEARRLAEEAAEARRLAE
TcDm25H2_000348700.1      EEAAEARRLAEEAAEARRLAEEAAEARRLAEEAEARRLAEEAAEARRLAEEAAEARRLAE
                          . ::  ** :            *:.*** *. :.*** *           .*:.*** *.

TcBrA4_0102720-RA         ASSVRRRASASSAEATCF---CFSVRRRASAASSVRRRASASSARRRASAAS--------
TcDm25H1_000366000.1      EAAEARRLAEEAAEARRLTEEAAEARRLAEEAAEARRLTEEAEARRLAEEEKAHFLAGAE
TcDm25H2_000348700.1      EAAEARRLAEEAAEARRLTEEAAEARRLAEEAAEARRLTEEAEARRLAEEEKAHFLAGAE
                           ::  ** : .:***  :   . ..** *. *:..** :. :.*** *.  .

TcBrA4_0102720-RA         --SAGDVL--------------------------------------------LLL-----
TcDm25H1_000366000.1      DVSGGEVYGVNLTFMNFLLSPENSDFLEREESIKRMRIVRLERDDYLILCRGLMLASAHF
TcDm25H2_000348700.1      DVSGGEVYGVNLTFMNFLLSPENSDFLEREESIKRMRIVRLERDDYLILCRGLMLASAHF
                            *.*:*                                             *:*

TcBrA4_0102720-RA         ------------------------------------------------------------
TcDm25H1_000366000.1      SDLREVTEPVKAETDSGWNDDDFDEDEMQFSNPCGANLAPSMSVGRVQMEEADDDEWGDW
TcDm25H2_000348700.1      SDLREVTEPVKAETDSGWNDDDFDEDEMQFSNPCGANLAPSMSVGRVQMEEADDDEWGDW
```

Case 2. Myosin head (motor domain). Chr08. Span difference: 675 bp

```
TcDm25H1_000562400.1      MSLDVGSLCFFKHPLESWVVSRVISRDARGYVVKTSDSERKCVGEVFSDVTGDRVAACRD
TcDm25H2_000583500.1      MSLDVGSLCFFKHPLESWVVSRVISRDARGYVVKTSDSERKCVGEVFSDVTGDRVAACRD
TcBrA4_0088840-RA         MSLDVGSLCFFKHPLESWVVSRVISRDARGYVVKTSDSERKCVGEVFSDVTGDRVAACRD
                          ************************************************************

TcDm25H1_000562400.1      DLLDEEPDDLLALTVLHDAPLLRCLYLRYFRNVIYTNIGAIVVAINPFNYTIPWYQDSQM
TcDm25H2_000583500.1      DLLDEEPDDLLALTVLHDAPLLRCLYLRYFRNVIYTNIGAIVVAINPFNYTIPWYQDSQM
TcBrA4_0088840-RA         DLLDEEPDDLLALTVLHDAPLLRCLYLRYFRNVIYTNIGAIVVAINPFNYTIPWYQDSQM
                          ************************************************************

TcDm25H1_000562400.1      VNYLQEGPVIEKNLPHSWAQAHNTYYDMISDRVNQCIIVSGESGAGKTETTKIVMKYLAQ
TcDm25H2_000583500.1      VNYLQEGPVIEKNLPHSWAQAHNTYYDMISDRVNQCIIVSGESGAGKTETTKIVMKYLAQ
TcBrA4_0088840-RA         VNYLQEGPVIEKNLPHSWAQAHNTYYDMISDRVNQCIIVSGESGAGKTETTKIVMKYLAQ
                          ************************************************************

TcDm25H1_000562400.1      VSCKEGTEDEKARSLEVGAKLDACSPILECFGNARTVRNDNSSRFGKFMRVKFNEKGQLV
TcDm25H2_000583500.1      VSCKEGTEDEKARSLEVGAKLDACSPILECFGNARTVRNDNSSRFGKFMRVKFNEKGQLV
TcBrA4_0088840-RA         VSCKEGTEDEKARSLEVGAKLDACSPILECFGNARTVRNDNSSRFGKFMRVKFNEKGQLV
                          ************************************************************

TcDm25H1_000562400.1      GAETTKYLLEKSRIVTAAEKERVYHSFYLLVRGTMGKTLWLEADTAYKSLNAGRCLQNSE
TcDm25H2_000583500.1      GAETTKYLLEKSRIVTAAEKERVYHSFYLLVRGTMGKTLWLEADTAYKSLNAGRCLQNSE
TcBrA4_0088840-RA         GAETTKYLLEKSRIVTAAEKERVYHSFYLLVRGTMGKTLWLEADTAYKSLNAGRCLQNSE
                          ************************************************************

TcDm25H1_000562400.1      YNTAKEYNEVIGAMTKIGISDEEVHSLWRCVGGILSLLNVTFDADGEGAQVQQATEKYLK
TcDm25H2_000583500.1      YNTAKEYNEVIGAMTKIGISDEEVHSLWRCVGGILSLLNVTFDADGEGAQVQQATEKYLK
TcBrA4_0088840-RA         YNTAKEYNEVIGAMTKIGISDEEVHSLWRCVGGILSLLNVTFDADGEGAQVQQATEKYLK
                          ************************************************************

TcDm25H1_000562400.1      DAVRLWRIEEATLRKEMVTTTLVVQKNETIKLLRPTLALDARDALVKALYDGLFGWLVDK
TcDm25H2_000583500.1      DAVRLWRIEEATLRKEMVTTTLVVQKNETIKLLRPTLALDARDALVKALYDGLFGWLVDK
TcBrA4_0088840-RA         DAVRLWRIEEATLRKEMVTTTLVVQKNETIKLLRPTLALDARDALVKALYDGLFGWLVDK
                          ************************************************************

TcDm25H1_000562400.1      CNRMCDVAVSGNWIGLLDIFGFEDFAKNSFEQLCINLTNETLQNHYNKYIFERDINECRE
TcDm25H2_000583500.1      CNRMCDVAVSGNWIGLLDIFGFEDFAKNSFEQLCINLTNETLQNHYNKYIFERDINECRE
TcBrA4_0088840-RA         CNRMCDVAVSGNWIGLLDIFGFEDFTKNSFEQLCINLTNETLQNHYNKYIFERDINECRE
                          *************************:**********************************

TcDm25H1_000562400.1      EGIDVTEVKCPDNSPCLQLIVGKSGIFALLNEECTLGKGSELAFLEKLDQAHTGKNSFFE
TcDm25H2_000583500.1      EGIDVTEVKCPDNSPCLQLIVGKSGIFALLNEECTLGKGSELAFLEKLDQAHTGKNSFFE
TcBrA4_0088840-RA         EGIDVTEVKCPDNSPCLQLIVGKSGIFALLNEECTLGKGSELAFLEKLDQAHTGKNSFFE
                          ************************************************************

TcDm25H1_000562400.1      KKKVSRDTFIIHHYAASVTYDVNGWLEKNRDTLKDGVKRMMRNSQDPLIREFLEAPLPPE
TcDm25H2_000583500.1      KKKVSRDTFIIHHYAASVTYDVNGWLEKNRDTLKDGVKRMMRNSQDPLIREFLEAPLPPE
TcBrA4_0088840-RA         KKKVSRDTFIIHHYAASVTYDVNGWLEKNRDTLKDGVKRMMRNSQDPLIREFLEAPLPPE
                          ************************************************************

TcDm25H1_000562400.1      TRGKRLTVGAVFRGQLDALMGVINTTNPHWIRCIKPHPAKKPLMFDGLQTMRQLESSGVL
TcDm25H2_000583500.1      TRGKRLTVGAVFRGQLDALMGVINTTNPHWIRCIKPHPAKKPLMFDGLQTMRQLESSGVL
TcBrA4_0088840-RA         TRGKRLTVGAVFRGQLDALMGVINTTNPHWIRCIKPHPAKKPLMFDGLQTMRQLESSGVL
                          ************************************************************

TcDm25H1_000562400.1      GTVKIRKAGYPVRNIFEKFNKRYNIILGAKAQGKSGRELAQMILIACGINSRAIAQLGKT
TcDm25H2_000583500.1      GTVKIRKAGYPVRNIFEKFNKRYNIILGAKAQGKSGRELAQMILIACGINSRAIAQLGKT
TcBrA4_0088840-RA         GTVKIRKAGYPVRNIFEKFNKRYNIILGAKAQGKSGRELAQMILIACGINSRAIAQLGKT
                          ************************************************************

TcDm25H1_000562400.1      KVFMKAEAFPIVERLRNESLLKLCLRLQSCGRAYLVRVYANREHCEQKCRRLAMLLTHEF
TcDm25H2_000583500.1      KVFMKAEAFPIVERLRNESLLKLCLRLQSCGRAYLVRVYANREHCEQKCRRLAMLLTHEF
TcBrA4_0088840-RA         KVFMKAEAFPIVERLRNESLLKLCLRLQSCGRAYLVRVYANREHCEQKCRRLAILLTHEF
                          *****************************************************:******
```

```
TcDm25H1_000562400.1      RVYMKRSVELREAKARWRKEQLALFSQRVAALYEECEGEKVQIHCEAIRLAQGLQIALKK
TcDm25H2_000583500.1      RVYMKRSVELREAKARWRKEQLALFSQRVAALYEECEGEKVQIHCEAIRLAQGLQIALKK
TcBrA4_0088840-RA         RVYMKRSVELREAKARWRKEQLALFSQRVAALYEECEGEKVQIHCEAIRLAQGLQIALKK
                          ************************************************************

TcDm25H1_000562400.1      HVELIKSWEVQKSTERRRLVDADLAARERMLQEATFYLEGLRCVFREEQRLFFTMLLGEL
TcDm25H2_000583500.1      HVELIKSWEVQKSTERRRLVDADLAARERMLQEATFYLEGLRCVFREEQRLFFTMLLGEL
TcBrA4_0088840-RA         HVELIKSWEVQKSTERRRLVDADLAARERMLQEATFYLEGLRCVFREEQRLFFTMLLGEL
                          ************************************************************

TcDm25H1_000562400.1      LDVEFGRRAQIRSVESEERSPLWRAYGLRHAMQQLEVLYREALMSHRCMEAMEFIQRDEI
TcDm25H2_000583500.1      LDVEFGRRAQIRSVESEERSPLWRAYGLRHAMQQLEVLYREALMSHRCMEAMEFIQRDEI
TcBrA4_0088840-RA         LDVEFGRRAQIRSVESEERSPLWRAYGLRHAMQQLEVLYREALMSHRCMEAMEFIQRDEI
                          ************************************************************

TcDm25H1_000562400.1      KERRALEQKNRSYWKSFKRLFRDKEGMATRMSGCREERERARVSQLMALHDVVAKEVRQQ
TcDm25H2_000583500.1      KERRALEQKNRSYWKSFKRLFRDKEGMATRMSGCREERERARVSQLMALHDVVAKEVRQQ
TcBrA4_0088840-RA         KERRALEQKNRSYWKSFKRLFRDKEGY---------------------------------
                          **************************

TcDm25H1_000562400.1      QGRSHSSHMTPTNFSTTTHDIGPDLQNHPRSPSGIDTQCSTTPHLLLLSGESRAQSETPF
TcDm25H2_000583500.1      QGRSHSSHMTPTNFSTTTHDIGPDLQNHPRSPSGIDTQCSTTPHLLLLSGESRAQSETPF
TcBrA4_0088840-RA         ------------------------------------------------------------


TcDm25H1_000562400.1      PAAFINGFVSTNRPMQFWGKRPKQGAKTPVHVSVVPTYDFSTSPMPSAVIDEKWGKKKLM
TcDm25H2_000583500.1      PAAFINGFVSTNRPMQFWGKRPKQGAKTPVHVSVVPTYDFSTSPMPSAVIDEKWGRKKLM
TcBrA4_0088840-RA         ------------------------------------------------------------


TcDm25H1_000562400.1      NNHDRARPSSPFCERASNLVSWPYAVATHASADRRLFPKLSAVISPSDVPRGAPPGKLHP
TcDm25H2_000583500.1      NNHDRARPSSPFCERASNLVSWPYAVATHASADRRLFPKLSAVISPSDVPRGAPPGKLHP
TcBrA4_0088840-RA         -----GQPECP------------------------------------DVGRNV-------
                               ..*..*                                    ** *..

TcDm25H1_000562400.1      SVAGANLDGTVQETVASGPRYKRSKRWA
TcDm25H2_000583500.1      SVAGANLDGTVQETVASGPRYKRSKRWA
TcBrA4_0088840-RA         ----------------------RERR--
                                                *..*
```

Case 3. DEAH box helicase. Chr28. Span difference: 2086 bp. Two CDS

```
TcDm25H1_001399700.1      MSYGEAYPTGDRMGALGANLHDVNWNEVQVVASQWNYYKPQQQRSDAEVAQWLRENRITI
TcDm25H2_001361500.1      MSYGEAYPTGDRMGALGANLHDVNWNEVQVVASQWNYYKPQQQRSDAEVAQWLRENRITI
TcBrA4_0000780-RA         MSYGEAYPTGDRMGALGANLHDVNWNEVQVVASQWNYYKPQQQRSDAEVAQWLRENRITI
                          ************************************************************

TcDm25H1_001399700.1      YGDRVPQPMLLFSDLVAPDSIHQSFIDLGYKEPTPIQSIAWPILLNSRDLVGVAKTGSGK
TcDm25H2_001361500.1      YGDRVPQPMLLFSDLVAPDSIHQSFIDLGYKEPTPIQSIAWPILLNSRDLVGVAKTGSGK
TcBrA4_0000780-RA         YGDRVPQPMLLFSDLVAPDSIHQSFIDLGYKEPTPIQSIAWPILLNSRDLVGVAKTGSGK
                          ************************************************************

TcDm25H1_001399700.1      TMAFMVPAALHIMAQPPVRPGDGPIALVLAPTRELAVQIEEETRKVLRRIPTIATTCLYG
TcDm25H2_001361500.1      TMAFMVPAALHIMAQPPVRPGDGPIALVLAPTRELAVQIEEETRKVLRRIPTIATTCLYG
TcBrA4_0000780-RA         TMAFMVPAALHIMAQPPVRPGDGPIALALAPTRELAVQIEEETRKVLRRIPTIATTCLYG
                          ***************************.********************************

TcDm25H1_001399700.1      GAPKGPQIRTLRAGVHVAIATPGRLIDLLEMRATNLLRVTYLVLDEADRMLDMGFEIQIR
TcDm25H2_001361500.1      GAPKGPQIRTLRAGVHVAIATPGRLIDLLEMRATNLLRVTYLVLDEADRMLDMGFEIQIR
TcBrA4_0000780-RA         GAPKGPQIRTLRAGVHVAIATPGRLIDLLEMRATNLLRVTYLVLDEADRMLDMGFEIQIR
                          ************************************************************

TcDm25H1_001399700.1      KICSQIRSDRQTLMFSATWPQEIRNLAASFQRDFIRVHVGSEDLVANNDVCQHVIVVEEY
TcDm25H2_001361500.1      KICSQIRSDRQTLMFSATWPQEIRNLAASFQRDFIRVHVGSEDLVANNDVCQHVIVVEEY
TcBrA4_0000780-RA         KICSQIRSDRQTLMFSATWPQEIRNLAASFQRDFIRVHVGSEDLVANNDVCQHVIVVEEY
                          ************************************************************

TcDm25H1_001399700.1      DKQRRLEEILQKLGRQRVLIFVKTKRTADSLHGSLRRILGGAVMAIHGDKEQSQRDYVLD
TcDm25H2_001361500.1      DKQRRLEEILQKLGRQRVLIFVKTKRTADSLHGSLRRILGGAVMAIHGDKEQSQRDYVLD
TcBrA4_0000780-RA         DKQRRLEEILQKLGRQRVLIFVKTKRTADSLHGSLRRILGGAVMAIHGDKEQSQRDYVLD
                          ************************************************************

TcDm25H1_001399700.1      RFRRDDRSVLVATDVAARGLDIKNLDVVINFDMPTNIEDYVHRIGRTGRAGQRGDAYSFV
TcDm25H2_001361500.1      RFRRDDRSVLVATDVAARGLDIKNLDVVINFDMPTNIEDYVHRIGRTGRAGQRGDAYSFV
TcBrA4_0000780-RA         RFRRDDRSVLVATDVAARGLDIKNLDVVINFDMPTNIEDYVHRIGM--------------
                          *********************************************

TcDm25H1_001399700.1      SGADPSKTIRDLVDLLRRANQEVPPELYEMMGRGGGGGGYRGGGGGGYRRGGGGGYRMGG
TcDm25H2_001361500.1      SGADPSKTIRDLVDLLRRANQEVPPELYEMMGRGGGGGGYRGGGGGGYRRGGGGGYRMGG
TcBrA4_0000780-RA         ------------------------------------------------------------


TcDm25H1_001399700.1      GGGGYRGGYDARLYAPYGSAVTAPVPSAPLLHAGGYPAAPRLPAGVGGGGGMYGVPPPPP
TcDm25H2_001361500.1      GGGGYRGGYDARLYAPYGSAVAAPVPSAPLLHAGGYPAAPRLPAGVGGGGGMYGVPPPPP
TcBrA4_0000780-RA         ------------------------------------------------------------


TcDm25H1_001399700.1      PPPPRVPISMPSAAPQWSQGPTSLAGEKRARLSPERRRSDSREKYRRHSPSDSRQRYRDS
TcDm25H2_001361500.1      PPPPRVPISMPSAAPQWSQGPTSLAGEKRARLSPERRRSDSREKYRRHSPSDSRQRYRDS
TcBrA4_0000780-RA         ------------------------------------------------------------


TcDm25H1_001399700.1      DDSYHRRHDSRERR
TcDm25H2_001361500.1      DDSYHRRHDSRERR
TcBrA4_0000780-RA         --------------
```

Case 4. endosomal trafficking protein. Chr15. Span difference: -3012 bp.

```
TcDm25H1_000909800.1      MDEVVLGVPQLAGCEAARRKPFEEYTSRYTVVKNSWKGKYMRIFCIGPRQVATINPQSIF
TcDm25H2_000928600.1      MDEVVLGVPQLAGCEAARRKPFEEYTSRYTVVKNSWKGKYMRIFCIGPRQVATINPQSIF
TcBrA4_0028840-RA         MDEVVLGVPQLAGCEAARRKPFEEYTSRYTVVKNSWKGKYMRIFCIGPRQVATINPQSIF
                          ************************************************************

TcDm25H1_000909800.1      RVTNNWEYNSHLVDVVATPGSPTDFTVTIGKIGKPETMSFHCNTAMERAELLTDVQRNRA
TcDm25H2_000928600.1      RVTNNWEYNSHLVDVVATPGSPTDFTVTIGKIGKPETMSFHCNTAMERAELLTDVQRNRA
TcBrA4_0028840-RA         RVTNNWEYNSHLVDVVATPGSPTDFTVTIGKIGKPETMSFHCNTAMERAELLTDVQRNRA
                          ************************************************************

TcDm25H1_000909800.1      KFDARYRQQVFNHVFDARKYSFYEEYRNCRIRITSIAVEQLSPEGNVVGEYLFMHIKGLT
TcDm25H2_000928600.1      KFDARYRQQVFNHVFDARKYSFYEEYRNCRIRITSIAVEQLSPEGNVVGEYLFMHIKGLT
TcBrA4_0028840-RA         KFDARYRQQVFNHVFDARKYSFYEEYRNCRIRITSIAVEQLSPEGNVVGEYLFMHIKGLT
                          ************************************************************

TcDm25H1_000909800.1      SILANPRTLILLYGPQLKMHLYEMEDCQRVVTLIEEFSRRFIGLPPLREVRRLTQQQFDS
TcDm25H2_000928600.1      SILANPRTLILLYGPQLKMHLYEMEDCQRVVTLIEEFSRRFIGLPPLREVRRLTQQQFDS
TcBrA4_0028840-RA         SILANPRTLILLYGPQLKMHLYEMEDCQRVVTLIEEFSRRFIGLPPLREVRRLTQQQFDS
                          ************************************************************

TcDm25H1_000909800.1      DRLGVDRAEMASLAKFPVSKWSSKHPDTPMRRILATTQKYLLELDATTYNPVSAFFFADI
TcDm25H2_000928600.1      DRLGVDRAEMVSLAKFPVSKWSSKHPDTPMRRILATTQKYLLELDATTYNPVSAFFFADI
TcBrA4_0028840-RA         DRLGVDRAEMASLAKFPVSKWSSKHPDTPMRRILATTQKYLLELDATTYNPVSAFFFADI
                          **********.*************************************************

TcDm25H1_000909800.1      YALIRSEEDEQRLMIQFKEPAITKVYTSPMRDALLAHLVDCCRASRNLNVCVIANLFDRG
TcDm25H2_000928600.1      YALIRSEEDEQRLMIQFKEPAITKVYTSPMRDALLAHLVDCCRASRNLNVCVIANLFDRG
TcBrA4_0028840-RA         YALIRSEEDEQRLMIQFKEPAITKVYTSPMRDALLAHLVDCCRASRNLNVCVIANLFDRG
                          ************************************************************

TcDm25H1_000909800.1      KRAAPCRTPIPEEIESTLLNCLIDPSKGGGPIAMTFPEVVEFFNANIEYSGLRLSENREG
TcDm25H2_000928600.1      KRAAPCRTPIPEEIESTLLNCLIDPSKGGGPIAMTFPEVVEFFNANIEYSGLRLSENREG
TcBrA4_0028840-RA         KRAAPCRTPIPEEIESTLLNCLIDPSKGGGPIAMTFPEVVEFFNANIEYSGLRLSENREG
                          ************************************************************

TcDm25H1_000909800.1      LFAENREKMIFSGLAALLNNFPVTDDPLVVVQQFYALRRLCVARIGFSSVAVVPSFMKNI
TcDm25H2_000928600.1      LFAENREKMIFSGLAALLNNFPVTDDPLVVVQQFYALRRLCVARIGFSSVAVVPSFMKNI
TcBrA4_0028840-RA         LFAENREKMIFSGLAALLNNFPVTDDPLVVVQQFYALRRLCVARIGFSSVAVVPSFMKNI
                          ************************************************************

TcDm25H1_000909800.1      EDVSMKALKMNHVAVSHAVVDLLGTLMTPHHDYYELAHEEANKNYILGQEQLVLRLLRML
TcDm25H2_000928600.1      EDVSMKALKMNHVAVSHAVVDLLGTLMTPHHDYYELAHEEANKNYILGQEQLVLRLLRML
TcBrA4_0028840-RA         EDVSMKALKMNHVAVSHAVVDLLGTLMTPHHDYYELAHEEANKNYILGQEQLVLRLLRML
                          ************************************************************

TcDm25H1_000909800.1      RDYATTESAALVVQALLDFFVYALCPPYCESTEAMLFTSVMKDLVDTTGKELFLLMQHGC
TcDm25H2_000928600.1      RDYATTESAALVVQALLDFFVYALCPPYCESTEAMLFTSVMKDLVDTTGKELFLLMQHGC
TcBrA4_0028840-RA         RDYATTESAALVVQALLDFFVYALCPPYCESTEAMLFTSVMKDLVDTTGKELFLLMQHGC
                          ************************************************************

TcDm25H1_000909800.1      NAISYSAGQLIRVIMEEGTGEQFRALQLAALSEGGIIGQLHLAIFSKNRELRDLARQLIA
TcDm25H2_000928600.1      NAISYSAGQLIRVIMEEGTGEQFRALQLAALSEGGIIGQLHLAIFSKNRELRDLARQLIA
TcBrA4_0028840-RA         NAISYSAGQLIRVIMEEGTGEQFRALQLAALSEGGIIGQLHLAIFSKNRELRDLARQLIA
                          ************************************************************

TcDm25H1_000909800.1      YWTYENTDMQDLMRNIFPPALLYYLQSSEEPPKDEMEEERQRNVVPMTSAFLESKLGWFK
TcDm25H2_000928600.1      YWTYENTDMQDLMRNIFPPALLYYLQSSEEPPKDEMEEERQRNVVPMTSAFLESKLGWFK
TcBrA4_0028840-RA         YWTYENTDMQDLMRNIFPPALLYYLQSSEEPPKDEMEEERQRNVVPMTSAFLESKLGWFK
                          ************************************************************

TcDm25H1_000909800.1      KRFHPSEVLSRGAAQNLSSSQGDGVFKRPRHVKVKTTLNWPMFFYEIKRDHLRPELIWNH
TcDm25H2_000928600.1      KRFHPSEVLSRGAAQNLSSSQGDGVFKRPRHVKVKTTLNWPMFFYEIKRDHLRPELIWNH
TcBrA4_0028840-RA         KRFHPSEVLSRGAAQNLSSSQGDGVFKRPRHVKVKTTLNWPMFFYEIKRDHLRPELIWNH
                          ************************************************************
```

```
TcDm25H1_000909800.1      TTRTELREALETEMRVLRLGMSLRHENPTSWNYREFEVRYPSLDDELRIGQHYPRLLFEM
TcDm25H2_000928600.1      TTRTELREALETEMRVLRLGMSLRHENPTSWNYREFEVRYPSLDDELRIGQHYPRLLFEM
TcBrA4_0028840-RA         TTRTELREALETEMRVLRLGMSLRHENPTSWNYREFEVRYPSLDDELRIGQHYPRLLFEM
                          ************************************************************

TcDm25H1_000909800.1      KDPAISRPKEFFNDMYHRFLLSQEPKTKMSCLHGMTILYEHYAADIGQFNDVEYIVRMLE
TcDm25H2_000928600.1      KDPAISRPKEFFNDMYHRFLLSQEPKTKMSCLHGMTILYEHYAADIGQFNDVEYIVRMLE
TcBrA4_0028840-RA         KDPAISRPKEFFNDMYHRFLLSQEPKTKMSCLHGMTILYEHYAADIGQFNDVEYIVRMLE
                          ************************************************************

TcDm25H1_000909800.1      TTFDPIFRDRLLIFILQLMRVRCNVKLFLDCDGLKPLVDLFTLAHLHVDRPQLRNATNAI
TcDm25H2_000928600.1      TTFDPIFRDRLLIFILQLMRVRCNVKLFLDCDGLKPLVDLFTLAHLHVDRPQLRNATNAI
TcBrA4_0028840-RA         TTFDPIFRDRLLIFILQLMRVRCNVKLFLDCDGLKPLVDLFTLAHLHVDRPQLRNATNAI
                          ************************************************************

TcDm25H1_000909800.1      ENNADTTDLQDQEKEWYYTRDGAKQGPISYIRLKHLYEEGEIKTDTKVWAQGLSGWKELK
TcDm25H2_000928600.1      ENNADTTDLQDQEKEWYYTRDGAKQGPISYIRLKHLYEEGEIKTDTKVWAQGLSGWKELK
TcBrA4_0028840-RA         ENNADTTDLQDQEKEWYYTRDGAKQGPISYIRLKHLYEEGEIKTDTKVWAQGLSGWKELK
                          ************************************************************

TcDm25H1_000909800.1      EVPQLRWGIMASKSNKLLTLTEVSCVILDILLLLCACFPSMDEHGAIMQPPPRVKRFLSS
TcDm25H2_000928600.1      EVPQLRWGIMASKSNKLLTLTEVSCVILDILLLLCACFPSMDEHGAIMQPPPRVKRFLSS
TcBrA4_0028840-RA         EVPQLRWGIMASKSNKLLTLTEVSCVILDILLLLCACFPSMDEHGAIMQPPPRVKRFLSS
                          ************************************************************

TcDm25H1_000909800.1      PQVLPHIVQLLLTFDPGLCARVHSLLYLIMEHNPLMSRFFLTGAFFFALMYIGSDVLALC
TcDm25H2_000928600.1      PQVLPHIVQLLLTFDPGLCARVHSLLYLIMEHNPLMSRFFLTGAFFFALMYIGSDVLALC
TcBrA4_0028840-RA         PQVLPHIVQLLLTFDPGLCARVHSLLYLIMEHNPLMSRFFLTGAFFFALMYIGSDVLALC
                          ************************************************************

TcDm25H1_000909800.1      RLLHLSHRRQAFQFKDENEIVRQSILSTMLPPALVCFLTNHGPEQFADVLLGEYENPEVI
TcDm25H2_000928600.1      RLLHLSHRRQAFQFKDENEILRQSILSTMLPPALVCFLTNHGPEQFADVLLGEYENPEVI
TcBrA4_0028840-RA         RLLHLSHRRQAFQFKDENEIVRQSILSAMLPPALVCFLTNHGPEQFADVLLGEYENPEVI
                          ********************:******:********************************

TcDm25H1_000909800.1      WGKDMRRYLVEK------------------------------------------------
TcDm25H2_000928600.1      WGKDMRRYLVEKIASHIADFTPRLLGNNRALYQYCPIVGVTYEPLRHELFCSQYYLRHFC
TcBrA4_0028840-RA         WGKDMRRYLVEKIASHIADFTPRLLGNNRALYQYCPIVGVTYEPLRHELFCSQYYLRHFC
                          ************

TcDm25H1_000909800.1      ------------------------------------------------------------
TcDm25H2_000928600.1      DELRYPNWPVDDPVNFLCEVLAAWRLELNKKSSGLTQDGCLEELEISDRSNLTLQVIRRG
TcBrA4_0028840-RA         DELRYPNWPVDDPVNFLCEVLAAWRLELNKKSSGLTQDGCLEELEISDRSNLTLQVIRRG


TcDm25H1_000909800.1      ------------------------------------------------------------
TcDm25H2_000928600.1      YFKLAAKYHPDKNPDGREKFERIQRAYEFLASETSVSDEPNPHIIALLLRTQSILFRRFS
TcBrA4_0028840-RA         YFKLAAKYHPDKNPDGREKFERIQRAYEFLASETSVSDEPNPHIIALLLRTQSILFRRFS


TcDm25H1_000909800.1      ------------------------------------------------------------
TcDm25H2_000928600.1      DVMKGYKYAGYSLLLKLIKMEFSDPEMLKKEVVLMEPATELCYFTVQNLPMNADELQEEG
TcBrA4_0028840-RA         DVMKGYKYAGYSLLLKLIKMEFSDPEMLKKEVVLMEPATELCYFTVQNLPMNADELQEEG


TcDm25H1_000909800.1      ------------------------------------------------------------
TcDm25H2_000928600.1      GIELLSGVTQRCFEILTPNATEELNQAKIVRHCMHTFRVAAGFADCRRHIVEEPVICHLA
TcBrA4_0028840-RA         GIELLSGVTQRCFEILTPNATEELNQAKIVRHCMHTFRVAAGFADCRRHIVEEPVICHLA


TcDm25H1_000909800.1      ------------------NCQSYC------------------------------------
TcDm25H2_000928600.1      AKGIAYEKAVGLSRACIQACQAFCVDEILQERVLKFGAIWHLLPFLFRYDYTVDENGLEL
TcBrA4_0028840-RA         AKGIAYEKAVGLSRACIQACQAFCVDEILQERVLKFGAIWHLLPFLFRYDYTVDENGLEL
                                             **::*
```

```
TcDm25H1_000909800.1          ------------------------------------------------------------
TcDm25H2_000928600.1          QEENHTQLFANRAAIYALRAIYALAGICPSDEYLLTKPNHEVICLLQRLLTPYIVRRMQL
TcBrA4_0028840-RA             QEENHTQLFANRAAIYALRAIYALAGICPSDEYLLTKPNDEVICLLQRLLTPYIVRRMQL


TcDm25H1_000909800.1          ------------------------------------------------------------
TcDm25H2_000928600.1          LPGDEKELLKLLNSNHNTPYLLWDNSTRQELLEMVKGNSKKCRDAGMFAEDLPVISSSVV
TcBrA4_0028840-RA             LPGDEKELLKLLNSNHNTPYLLWDNSTRQELLEMVKGNSKKCRDAGMFAEDLPVISSSVV


TcDm25H1_000909800.1          ----------------RLY-----------------------------------------
TcDm25H2_000928600.1          NYSLHADELVIGGVFVRVYNEQPNFAIEEPVAFCAAMIKFLEQQLTSNTTIGVLLTLEAL
TcBrA4_0028840-RA             NYSLHADELVIGGVFVRVYNEQPNFAIEEPVAFCAAMIKFLEQQLTSNTTTGVLLTLEAL
                                              *:*

TcDm25H1_000909800.1          ------------------------------------------------------------
TcDm25H2_000928600.1          KHLLIAYATAGVANTLKHHVGILIKVLLYEDTAITVKLMELLEKVALHHKCLEAIGNIDS
TcBrA4_0028840-RA             KHLLIAYATAGVANTLKHHVGILIKVLLYEDTAITVKLMELLEKVALHHKCLEAIGKIDS


TcDm25H1_000909800.1          ------------------------------------------------------------
TcDm25H2_000928600.1          AVAYIILALHRGGEVVESRCLTFLRVALADRGVAQQALDRGLYVVLLRILGTSTSPECRE
TcBrA4_0028840-RA             AVAYIILALHRGGEVVETRCLTFLRVALADRGVAQQALDRGLYVVLLRILGTSTSPECRE


TcDm25H1_000909800.1          ------------------------------------------------------------
TcDm25H2_000928600.1          DVCSSLAKACSDKLCGPKVFLRASKLLPSVMLETMNENTTNACQLFDTWQETPELMWTKE
TcBrA4_0028840-RA             DVCSSLAKACSDKLCGPKVFLRASKLLPSVMLETMNENTTNACQLFDTWQETPELMWTKE


TcDm25H1_000909800.1          ------------------------------------------------------------
TcDm25H2_000928600.1          RRSRFVEICLACQSDIVTTLQQDPTAYWKIPENILTERNKEMQIGGVYLERYMNQSGWIV
TcBrA4_0028840-RA             RRSRFVEICLACQSDIVTTLQQDPTAYWKIPENILTERNKEMQIGGVYLERYMNQSGWIV


TcDm25H1_000909800.1          ------------------------------------------------------------
TcDm25H2_000928600.1          RKPKEFLTALLERFVEESGRATEEKNAEMISLVADAGVRLLQTTPTVADYVVSLGYAQKL
TcBrA4_0028840-RA             RKPKEFLTALLERFVEESGRATEEKNAEMISLVADAGVRLLQTTPTVADYVVSLGYAQKL


TcDm25H1_000909800.1          ------------------------------------------------------------
TcDm25H2_000928600.1          FKLLELGDKVIAENALKWVHEICASRLCVESLGNFDPVFFLLVCLRAQFRQLPLIMDTMN
TcBrA4_0028840-RA             FKLLELGDKVIAENALKWVHEICASRLCVESLGNFDPVFFLLVFLRAQFRQLPLIMDTMN


TcDm25H1_000909800.1          ------------------------------------------------------------
TcDm25H2_000928600.1          RLMSHSSERANMIRLALRNQLPQRLLELLEDGITSENCGEQSPAAVRALIIKVLKTMVAV
TcBrA4_0028840-RA             RLMSHSSERANMIRLALRNQLPQRLLELLEDGITSENCGEQSPAAVRALIIKVLKTMVAV


TcDm25H1_000909800.1          --------------------------------ATTFG-----------------------
TcDm25H2_000928600.1          QDPLHGPQLEAILADSKVWVKYKNQSHDLFLSNTRFGGYLEGAYQQNQMLLSYAAAPLSD
TcBrA4_0028840-RA             QDPLHGPQLEAILADSKVWVKYKNQSHDLFLSNTRFGGYLEGAYQQNQMLLSYAAAPLSD
                                                               * **

TcDm25H1_000909800.1          ----------
TcDm25H2_000928600.1          EGSGQEPPPV
TcBrA4_0028840-RA             EGSGQEPPPV
```

Case 5. Protein kinase. Chr02. Span difference: -669 bp.

```
TcDm25H1_000121700.1      ------------------------------------------------------------
TcDm25H2_000111700.1      ------------------------------------------------------------
TcBrA4_0063370-RA         MPNPLVSDSSNTSSYSIDMDVNAMRSSFYNPLKTLIEQTILSFGYTSLKELAEIPIQDKM

TcDm25H1_000121700.1      ------------------------------------------------------------
TcDm25H2_000111700.1      ------------------------------------------------------------
TcBrA4_0063370-RA         SKSPTSGGNSRSGACCQKCKIYEKLLKKLATLLLVELDEDGGTSTNGAWPGLLSLEKPQG


TcDm25H1_000121700.1      ------------------------------------------------------------
TcDm25H2_000111700.1      ------------------------------------------------------------
TcBrA4_0063370-RA         MGFSGLVAGNQSLSGSRGSGASPKHDGATIPMLSLLTSPFDSFSSVTLMSTTPLTAPSYS


TcDm25H1_000121700.1      -------------------------------------------MLVSNRETAKADLMQRG
TcDm25H2_000111700.1      -------------------------------------------MLVSNRETAKADLMQRG
TcBrA4_0063370-RA         ARGSVGNWLKGFVAFAAPGKKSESNSFSDGDDYTRTSNSLTPAILVSNRETAKADLMQRG
                                                                     :****************

TcDm25H1_000121700.1      KNVKLARQAAVHVAHGTSYSIARARGWDANPRVKETSRTIIARKNQKKMINEYVLLRKIG
TcDm25H2_000111700.1      KNVKLARQAAVHVAHGTSYSIARARGWDANPRVKETSRTIIARKNQKKMINEYVLLRKIG
TcBrA4_0063370-RA         KNVKLARQAAVHVAHGTSYSIARARGWDANPRVKETSRTIIARKNQKKMINEYVLLRKIG
                          ************************************************************

TcDm25H1_000121700.1      QGSTGYVVLVQECESKELFAMKIVRLGNKIDWRRVNAIRSEITVLKAVAHPNLVRLHEVI
TcDm25H2_000111700.1      QGSTGYVVLVQECESKELFAMKIVRLGNKIDWRRVNAIRSEITVLKAVAHPNLVRLHEVI
TcBrA4_0063370-RA         QGSTGYVVLVQECESKELFAMKIVRLGNKIDWRRVNAIRSEITVLKAVAHPNLVRLHEVI
                          ************************************************************

TcDm25H1_000121700.1      GDKSHNTIFLILQYISGGSIAKTLSSVTIATIPEAKLRCYTVQILSALSHLHSNGIFHRD
TcDm25H2_000111700.1      GDKSHNTIFLILQYISGGSIAKTLSSVTIATIPEAKLRCYTVQILSALSHLHSNGIFHRD
TcBrA4_0063370-RA         GDKSHNTIFLILQYISGGSIAKTLSSVTIATIPEAKLRCYTVQILSALSHLHSKGIFHRD
                          *****************************************************:******

TcDm25H1_000121700.1      IKPENILIDKEERIYLADFGVSAISTANGVHGMEGTPAFMAPEVFTGNFELIGELVDVWA
TcDm25H2_000111700.1      IKPENILIDKEERIYLADFGVSAISTANGVHGMEGTPAFMAPEVFTGNFELIGELVDVWA
TcBrA4_0063370-RA         IKPENILIDKEERIYLADFGVSAISTANGVHGMEGTPAFMAPEVFTGNFELIGELVDVWA
                          ************************************************************

TcDm25H1_000121700.1      LGVTLYQLMYGFLPFQALTYFEMVRRIVNDPVTFPDKVQGDESERADLGDFSYLDEIEGF
TcDm25H2_000111700.1      LGVTLYQLMYGFLPFQALTYFEMVRRIVNDPVTFPDKVQGDESERADLGDFSYLDEIEGF
TcBrA4_0063370-RA         LGVTLYQLMYGFLPFQALTYFEMVRRIVNDPVTFPDKVQGDESERADLGDFSYLDEIEGF
                          ************************************************************

TcDm25H1_000121700.1      MNTDTYLGFHYVGEDDEDEPEKEKYHNDRDEVTPSAVHSSPEFKELIQGVLCKDPHSRWN
TcDm25H2_000111700.1      MNTDTYLGFHYVGEDDEDEPEKEKYHNDRDEVAPSAVHSSPEFKELIQGVLCKDPHSRWN
TcBrA4_0063370-RA         MNTDTYLGFHYVGEDDEDEPEKEKYHNDRDEVTPSAVHSSPEFKELIQGVLCKDPHSRWN
                          ********************************:***************************

TcDm25H1_000121700.1      LRRIWESAWLRDALESGRRITMTGSPTPRYGEVCFPRALSPSAMTRPAVENNSTNAPITK
TcDm25H2_000111700.1      LRRIWESAWLRDALESGRRITMTGSPTPRYGEVCFPRALSPSAMTRPAVENNSTNAPITK
TcBrA4_0063370-RA         LRRIWESAWLRDALESGRRITMTGSPTPRYGEVCFPRALSPSAMTRPAVENNSTNAPITK
                          ************************************************************

TcDm25H1_000121700.1      LNPNVAVKFYAVSNQTHNFPTNKINNQINTENTPINKTPTPIFKSNSKVRVNIDTAKESS
TcDm25H2_000111700.1      LNPNVAVKFYAVSNQTHNFPTNKINNQINTENTPINKTPTPIFKSNSKVRVNIDTAKESS
TcBrA4_0063370-RA         LNPNVAVKFYAVSNQTHNSPTNKINNQINTENTPINKTPTPIFKSNSKVRVNIDTAKESS
                          ****************** *****************************************

TcDm25H1_000121700.1      SVRQLKADEECARPRASLWKRILSGTWRRKKKVLPEGQS
TcDm25H2_000111700.1      SVRQLKADEECARPRASLWKRILSGTWRRKKKVLPEGQS
TcBrA4_0063370-RA         SVRQLKADEECARPRASLWKRILSGTWRRKKKVLPEGQS
                          ***************************************
```

Case 6. hypothetical protein conserved. Chr03. Span difference: -411 bp.

```
TcDm25H2_000192900.1      MPIKAKPVDPGRARLAALGQWNRQRRTTSTGPNSAKRRGSGSWANSTGSSTSTSSSEGSR
TcDm25H1_000197600.1      ------------------------------------------------------------
TcBrA4_0085270-RA         MPIKAKPVDPGRARLAALGQWNRQRRTTSTGPNSAKRRGSGSWANSTGSSTSTSSSEGSR


TcDm25H2_000192900.1      TGRRGRRRLYNQNGKREGNGNGGTPAFLTSSRTVDSDSGTSNSDPELYRAARERTERFLY
TcDm25H1_000197600.1      ------------------------------------------------------------
TcBrA4_0085270-RA         TGRRGRRRLYNQNGKREGNGNGGTPAFLTSSRTVDSDSGTSNSDPELYRAARERTERFLY


TcDm25H2_000192900.1      PNGRSGINVSSSNHGAVMEARGEATSNAENISLNASLAMAPVFSGLQSQNQKKESDNKRS
TcDm25H1_000197600.1      -----------------MEARGEATSNAENISLNASLPMAPVFSGLQSQNQKKESDNKRS
TcBrA4_0085270-RA         PNGRSGINVSSSNHGAVMEARGEATSNAENISLNASLPMAPVFSGLQSQNQKKESDNKRS
                                           ********************.**********************

TcDm25H2_000192900.1      DANLESSRKFFPPPSPAVVPLVVQEALRGLQGTKDSASEREFARLMDLFAADVQKEREQE
TcDm25H1_000197600.1      DANLESSRKFFPPPSPAVVPLVVQEALRGLQGTKDSASEREFARLMDLFAADVQKEREQE
TcBrA4_0085270-RA         DANLESSRKFFPPPSPAVVPLVVQEALRGLQGTKDSASEREFARLMDLFAADVQKEREQE
                          ************************************************************

TcDm25H2_000192900.1      KELQQEKELEVDTMDTVSSMMAKNQNQKPAQGPWEGTYKLYTSPPHTFDELQERALLQLY
TcDm25H1_000197600.1      KELQQEKELEVDTMDTVSSMMAKNQNQKPAQGPWDGTYKLYTSPPHTFDELQERALLQLY
TcBrA4_0085270-RA         KELQQEKELEVDTMDTVSSMMAKNQNQKPAQGPWDGTYKLYTSPPHTFDELQERALLQLY
                          **********************************:*************************

TcDm25H2_000192900.1      AANEENKDSGYFIATDHMAEAFTLAALEATRNEFPGESDAASGASGALNDNNEKFRPIGA
TcDm25H1_000197600.1      AANEENKDSGYFIATDHMAEAFTLAALEATRNEFPGESDAASGASGALNDNNEKFRPIGA
TcBrA4_0085270-RA         AANEENKDSGYFIATDHMAEAFTLAALEATRNEFPGESDAASGASGALNDNNEKFRPIGA
                          ************************************************************

TcDm25H2_000192900.1      PPSLMEIVAIERARRNARAAFQKRREGEEGVTIDRNTEALLSMDTEILAATAEENLNRFL
TcDm25H1_000197600.1      PPSLMEIVAIERARRNARAAFQKRREGEEGVTIDRNTEALLSMDTEILAATAEENLNRFL
TcBrA4_0085270-RA         PPSLMEIVAIERARRNARAAFQKRREGEEGVTIDRNTEALLSMDTEILAATAEENLNRFL
                          ************************************************************

TcDm25H2_000192900.1      LAVRCIENAVRNRRVTDALGRFLFAHHKPFLRTTRGAGSSADAIREFPHEAFAVYERYGH
TcDm25H1_000197600.1      LAVRCIENAVRNRRVTDALGRFLFAHHKPFLRTTRGAGSSADAIREFPHEAFAVYERYGH
TcBrA4_0085270-RA         LAVRCIENAVRNRRVTDALGRFLFAHHKPFLRTTRGAGSSADAIREFPHEAFAVYERYGH
                          ************************************************************

TcDm25H2_000192900.1      Y---------------FNMEEFVLSLFDVNMNGEENNNVSDAEDVSMDVLSYPAWRLLQS
TcDm25H1_000197600.1      YVGKFITELLNRNVPKFNMEEFVLSLFDVNMNGEENNNVSDAEDVSMDVLSYPAWRLLQS
TcBrA4_0085270-RA         YVGKFITELLNRNVPKFNMEEFVLSLFDVNMNGEENNNVSDAEDVSMDVLSYPAWRLLQS
                          *               ********************************************

TcDm25H2_000192900.1      ISSFNEFCTFMDDFIAEEYGVEKVVAHDNESTNGDAGVKASRYEGDGVVVVAGARGVRAL
TcDm25H1_000197600.1      ISSFNEFCTFMDDFIAEEYGVEKVVAHDNESTNGDAGVKASRYEGDGVVVVAGARGVRAL
TcBrA4_0085270-RA         ISSFNEFCTFMDDFIAEEYGVEKVVAHDNESTNGDAGVKASRYEGDGVVVVAGARGVRAL
                          ************************************************************

TcDm25H2_000192900.1      LTKSKAKLPSARASSTCDASQQMRVPNEGAHESNDEFLFLPSEGGATETPLSSPPRESQA
TcDm25H1_000197600.1      LTKSKAKLPSARASSTCDASQQMRVPNEGAHESNDEFLFLPSEGGATETPLSSPPRESQA
TcBrA4_0085270-RA         LTKSKAKLPSARASSTCDASQQMRVPNEGAHESNDEFLFLPSEGGATETPLSSPPRESQA
                          ************************************************************

TcDm25H2_000192900.1      HGDRSAAEFSTRTDSAPTVLSNSHPALSGSSRTPFGNSLTPPPATSLQDPRPSKNSLSTA
TcDm25H1_000197600.1      HGDRSAAEFSTRTDSAPTVLSNSHPALSGSSRTPFGNSLTPPPATSLQDPRPSKNSLSTA
TcBrA4_0085270-RA         HGDRSAAEFSTRTDSAPTVLSNSHPALSGSSRTPFGNSLTPPPATSLQDPRPSKNSLSTA
                          ************************************************************

TcDm25H2_000192900.1      NSLPQLPHGSRRIEAVRGRNLPPLSAAGGKEAETMFSTAPLKLSLGSEANSVPPGGFKAE
TcDm25H1_000197600.1      NSLPQLPHGSRRIEAVRGRNLPPLSAAGGKEAETMFSTAPLKLSLGSEANSVPPGGFKAE
TcBrA4_0085270-RA         NSLPQLPHGSRRIEAVRGRNLPPLSAAGGKEAETMFSTAPLKLSLGSEANSVPPGGFKAE
```

```
                              ************************************************************

TcDm25H2_000192900.1      PVTTERTVQGTDSSGKIDNVSGRQAPSSSIGISYGHKKKSKDSRSRSGARTGAETTTTTR
TcDm25H1_000197600.1      PVTTERTVQGTDSSGKIDNVSGRQAPSSSIGISYGHKKKSKDSRSRSGARTGAETTTTTR
TcBrA4_0085270-RA         PVKTERTVQGTDSSGKIDNVSGRQAPSSSIGISYGHKKKSKDSRSRSGARTGAETTTTTR
                          **.*********************************************************

TcDm25H2_000192900.1      KARGKKLKSPQDT
TcDm25H1_000197600.1      KARGKKLKSPQDT
TcBrA4_0085270-RA         KARGKKLKSPQDT
                          *************
```
