# Supplementary material for: A phased genome assembly of a Colombian Trypanosoma cruzi TcI strain and the evolution of gene families
Source: Sci Rep. 2024 Jan 24;14:2054. doi: 10.1038/s41598-024-52449-x (PMC10808112; doi:10.1038/s41598-024-52449-x)

Supplementary materials for:

A phased genome assembly of a Colombian *Trypanosoma cruzi* TcI strain and the evolution of gene families

Maria Camila Hoyos Sanchez[1,3], Hader Sebastian Ospina Zapata[2], Brayhan Dario Suarez[2], Carlos Ospina[2], Hamilton Julian Barbosa[2], Julio Cesar Carranza Martinez[2], Gustavo Adolfo Vallejo[2], Daniel Urrea Montes[2], Jorge Duitama[1,*]

1 Systems and Computing Engineering Department, Universidad de los Andes, Bogotá, Colombia.

2 Laboratorio de Investigaciones en Parasitología Tropical (LIPT), Universidad del Tolima, Ibagué, Colombia.

3 School of Veterinary Medicine, Texas Tech University, Amarillo, TX 79106, USA

*Corresponding author: ja.duitama@uniandes.edu.co

SUPPLEMENTARY FILES

Supplementary File 1. Examples of alignments of genes with extreme length differences between the Dm25 copies and the Brazil A4 copy.
Supplementary File 2. Combined genome assembly of the Dm25 strain in gzip compressed fasta format.
Supplementary File 3. Companion gene annotation for the combined genome assembly of the Dm25 strain in gzip compressed GFF3 format.

SUPPLEMENTARY TABLES (provided in a separate Excel file)

Supplementary Table 1. *T. cruzi* assemblies sequenced with long reads technologies or chromosome-level assemblies available in TriTrypDB to date.
Supplementary Table 2. Average read depth per contig after mapping raw reads to the H2 haplotype reconstruction
Supplementary Table 3. Distribution of families of repetitive elements in TcDm25
Supplementary Table 4. Locations of homologs in T. cruzi genome assemblies for tandem arrays of protein kinases (PKS) and histone core families (HIS)
Supplementary Table 5. Illumina-sequenced *T. cruzi* genomes downloaded from the TriTrypDB-48 database.

SUPPLEMENTARY FIGURES

Supplementary Figure 1. Basic statistics of the haplotypes making the *T. cruzi* Dm25 assembly A) Total length. B) Median contig length (N50) C) Distribution of hits for 130 genes conserved in Euglenozoa.

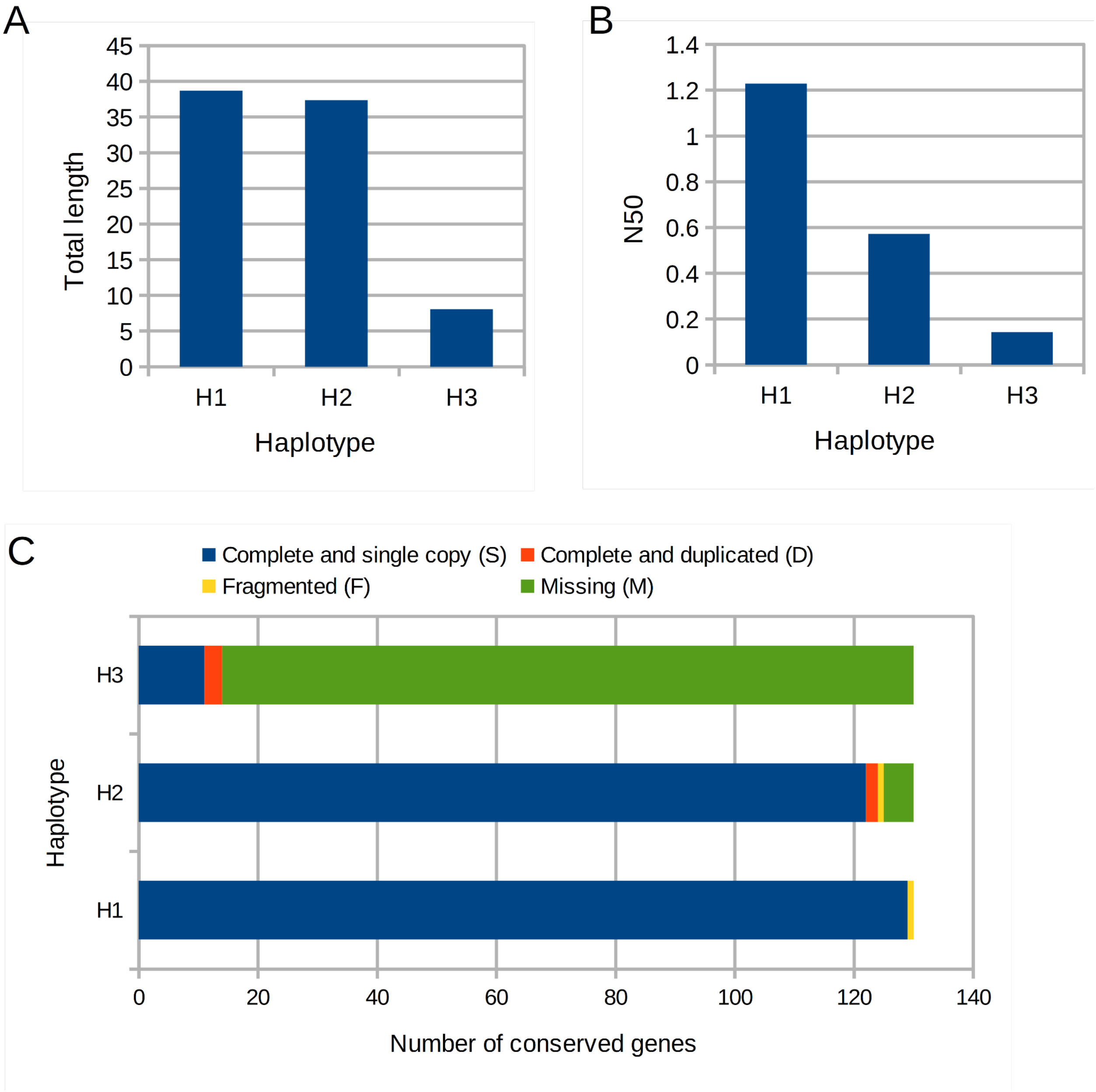

Supplementary Figure 2. Relative allele dosages in sites having more than one allele called from reads aligned to the contigs of the H1 assembly.

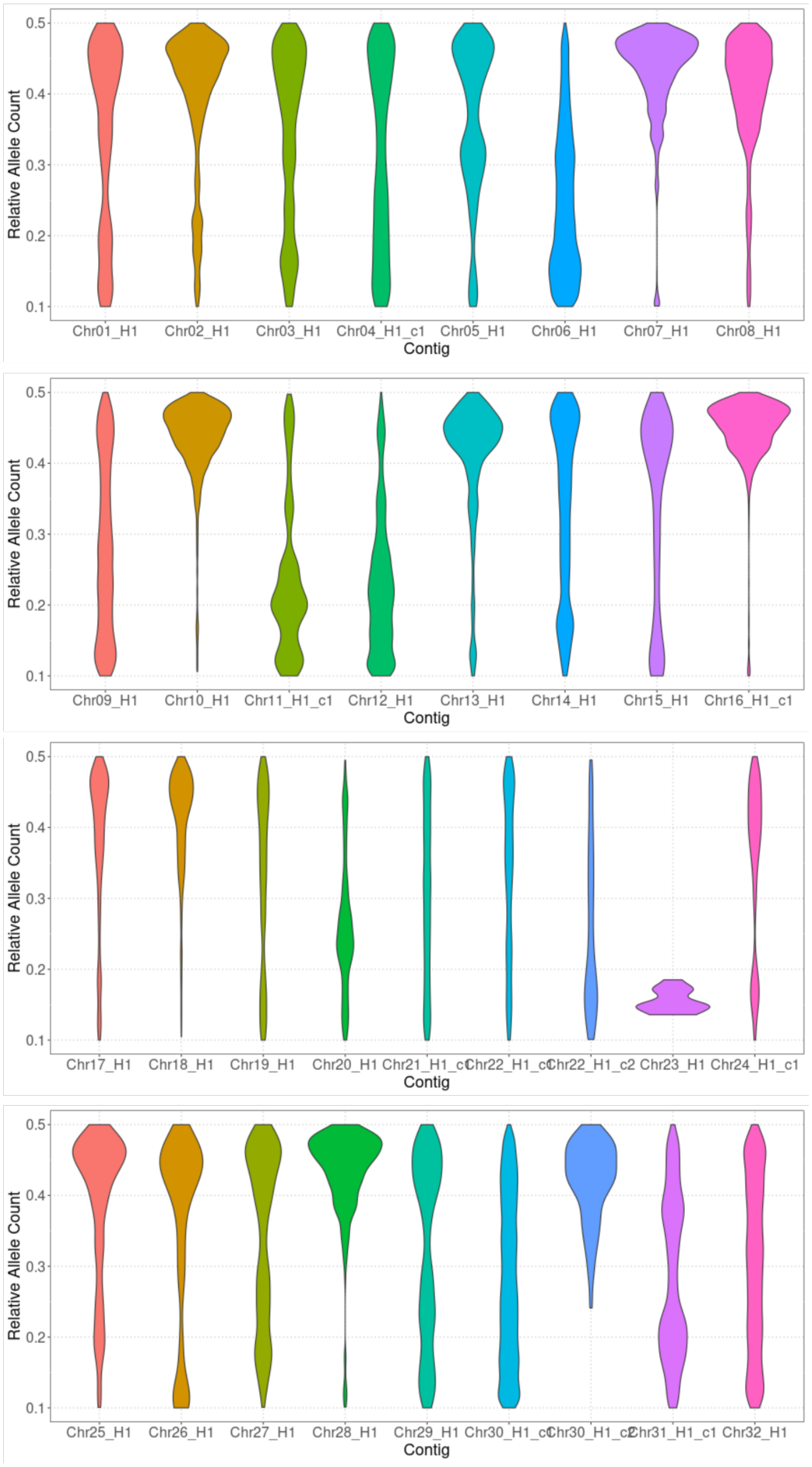

Supplementary Figure 3. Assembly of the maxicircle of the T. cruzi strain Dm25. Protein coding genes in the conserved region (CR) are shown in the external red rectangles. The divergent region is divided into the P5 region (purple) and the P12 region (blue). Conserved elements (CE) across the P12 region are shown as red rectangles. The central histogram shows the GC-skew, positive values are in red and negative values are shown in blue (windows size 100 bp). Internal bands show the homology relationships making up the P5 and P12 variable regions.

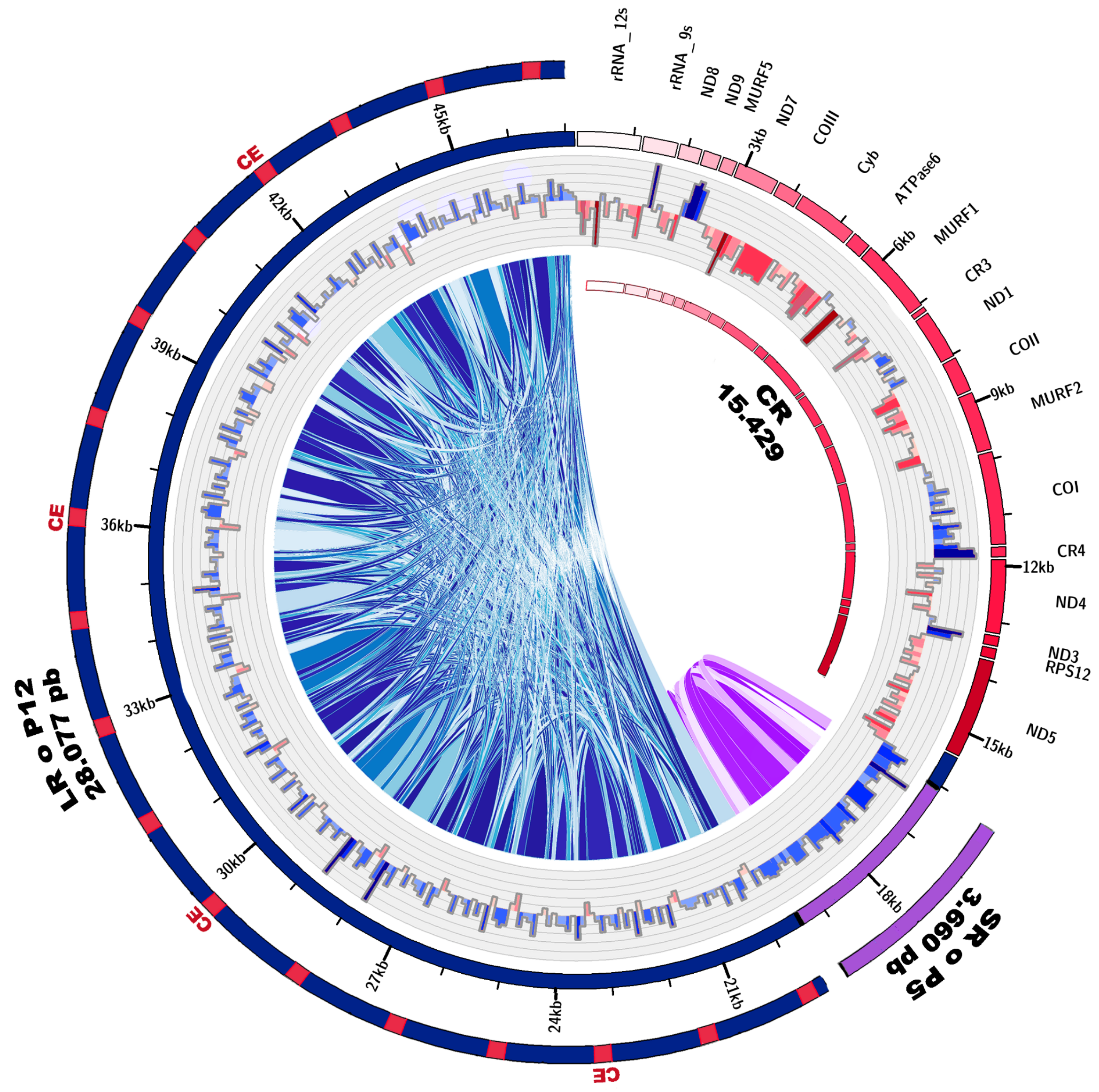

Supplementary Figure 4. Dotplot between the contig assigned to chromosome 31 in the H1 assembly and the three contigs assigned to the same chromosome in the H2 assembly.

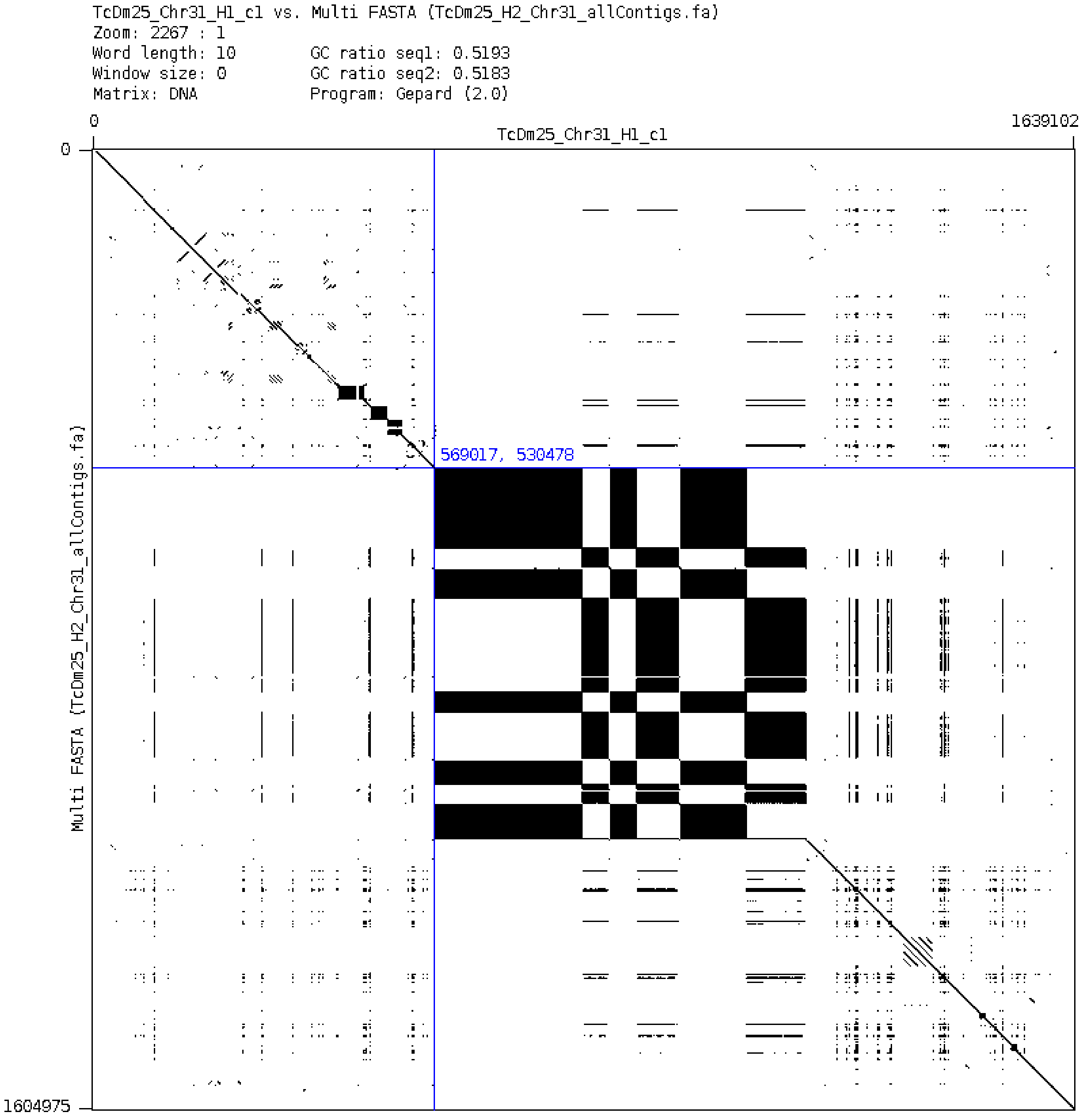

Supplementary Figure 5. Statistics of genes annotated in the TcDm25 assembly. **A-B**. Length distribution for genes and proteins annotated in the two haplotypes of TcDm25, and in the Brazil A4 strain. **C**. Number of copies of the six main gene families, compared with those reported in previous studies.

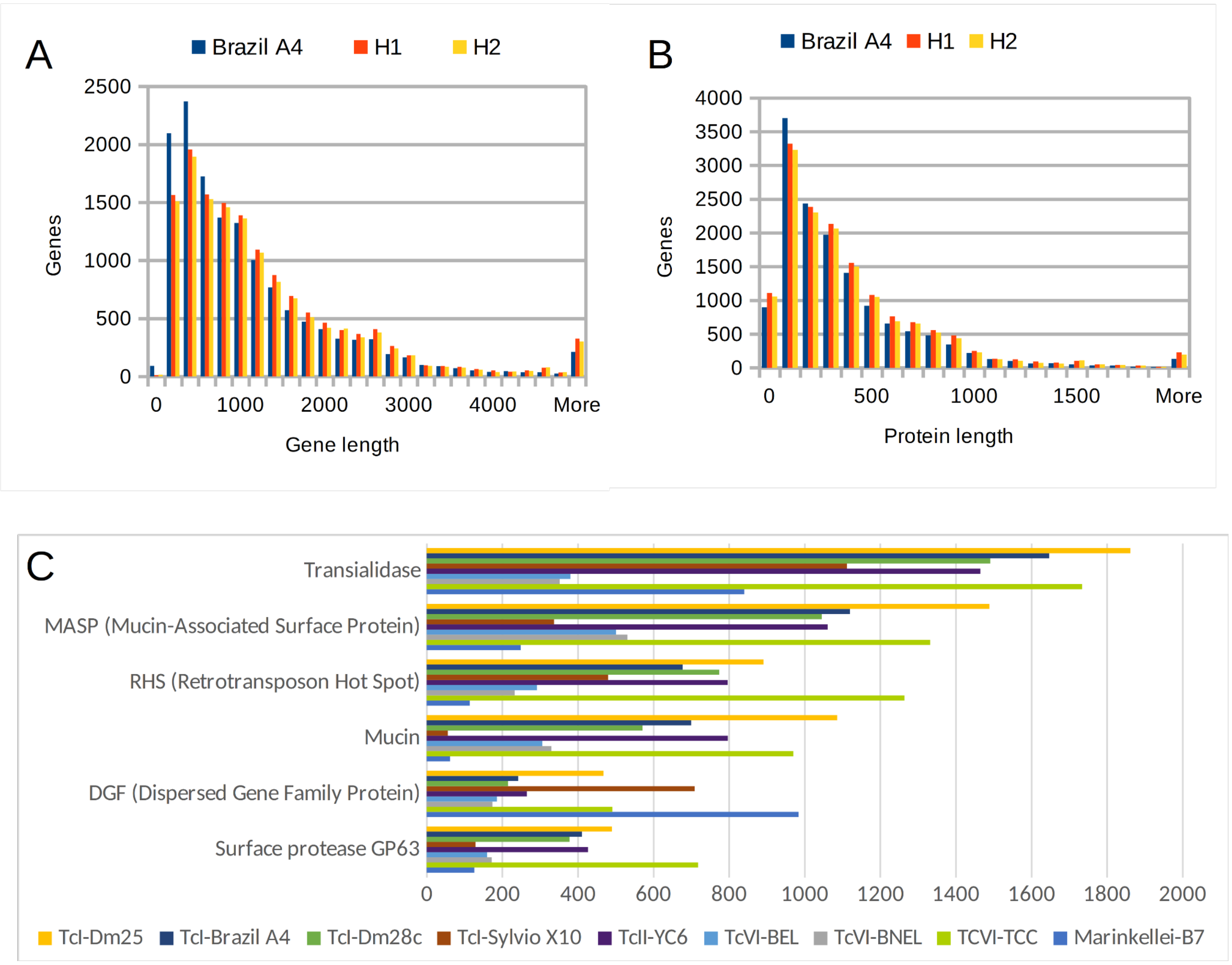

Supplementary Figure 6. Alignments of the contig assigned to chromosome 32 of the Dm25 haplotype H1 assembly against the contig c2 assigned to the same chromosome in the Dm25 H2 assembly, and against the homologous contigs that were found in other genome assemblies. The blue coordinates indicate the start of the tandem array of protein kinases (end in the case of Berenice). The numbers besides the alignments of protein kinases indicate the number of copies annotated in each contig. BEL: Brener Esmeraldo-Like. Rc. Reverse complement.

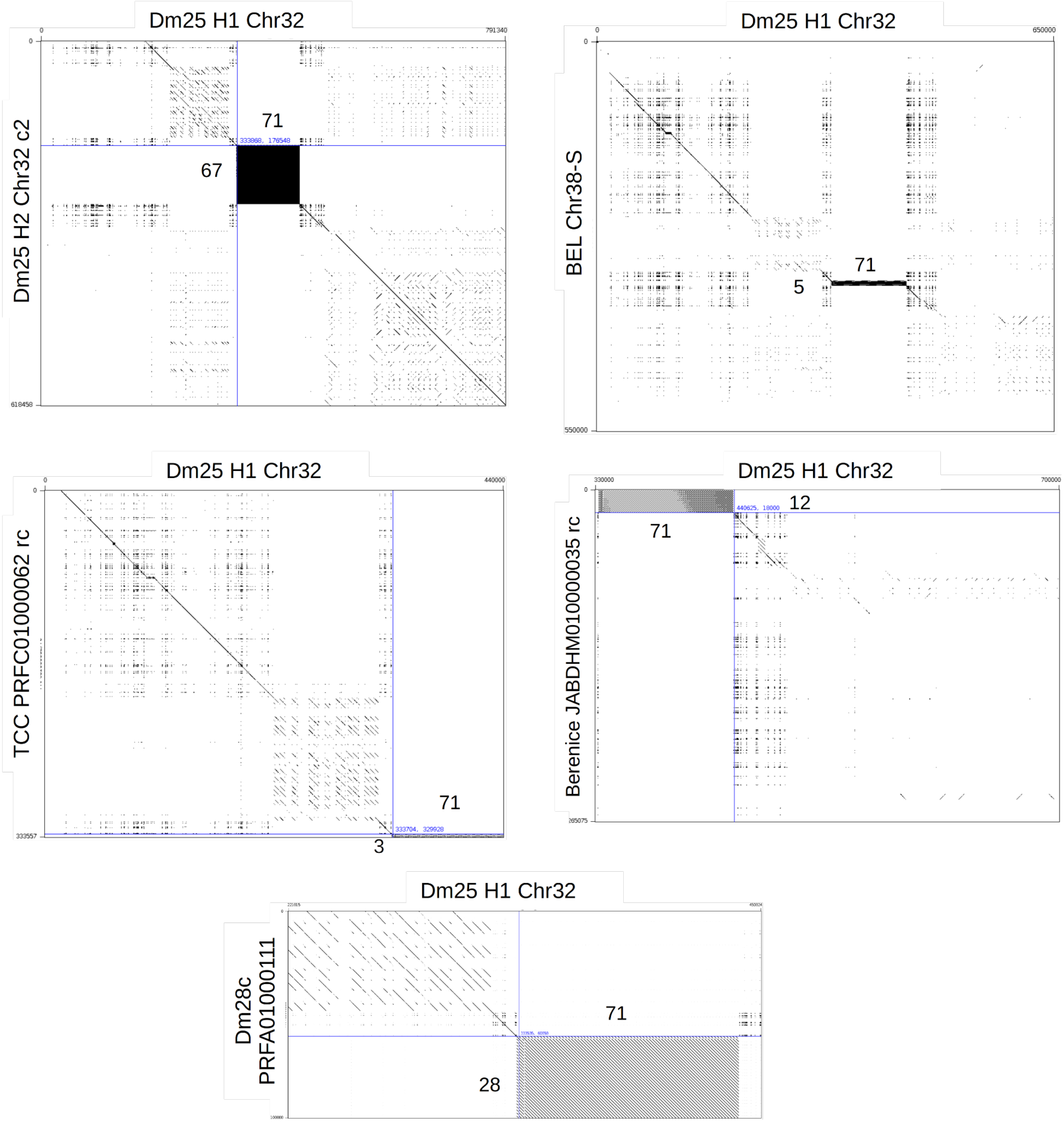

Supplementary Figure 7. Alignments of the contig assigned to chromosome 4 in the Dm25 haplotype H1 assembly against the contig c2 assigned to the same chromosome in the Dm25 H2, and to the homologous contigs that were found in other genome assemblies. The blue coordinates indicate the start of the tandem array of histone core proteins (end in the case at the bottom of the figure). The numbers besides the alignments of histones indicate the number of copies annotated in each contig. BEL: Brener Esmeraldo-Like.

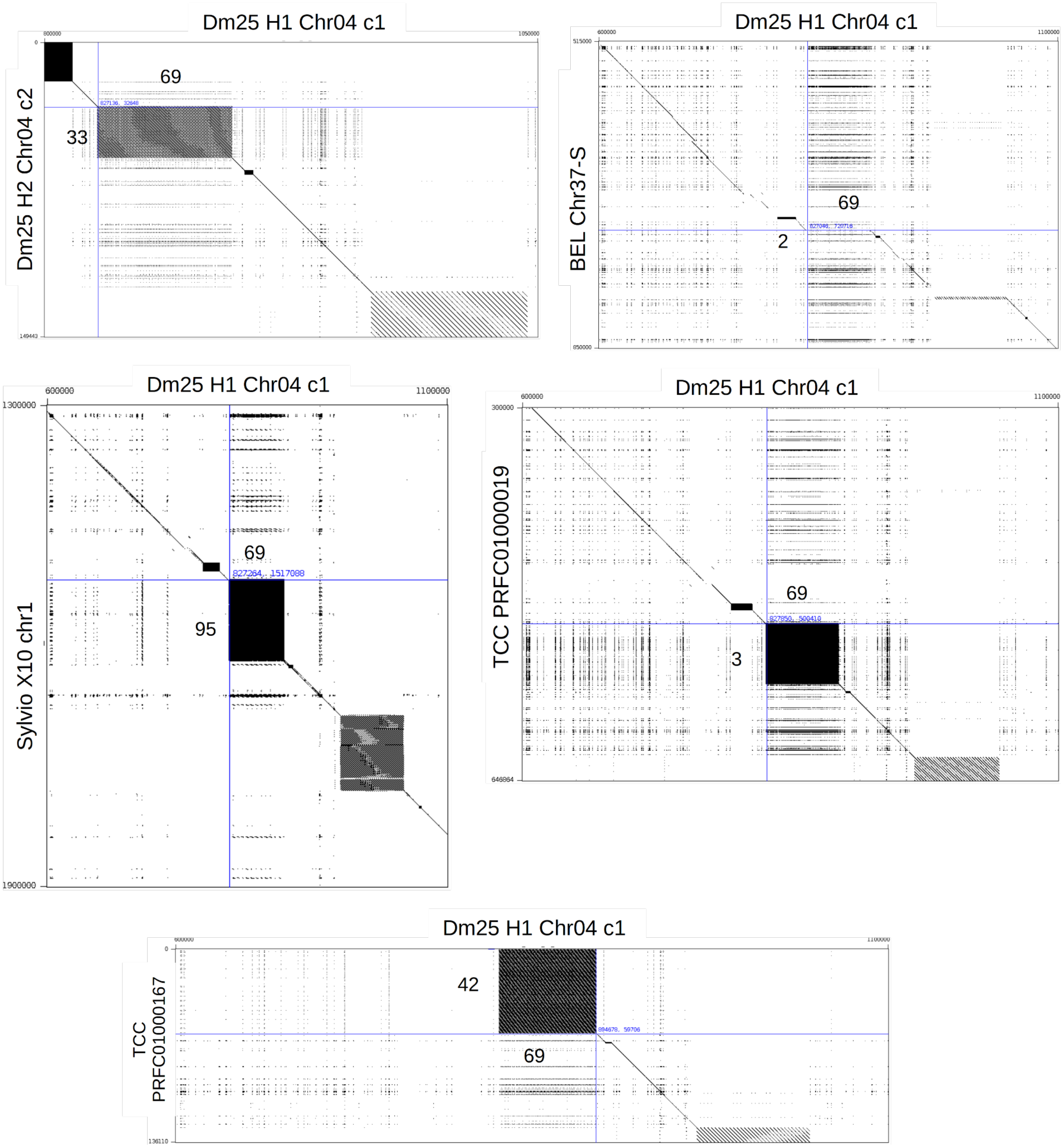

Supplementary Figure 8. Baseline information of Illumina reads obtained in Triptryp (DTU I: blue color, DTU II: green color, DTU III: gray color, DTU IV: orange, DTU V: yellow, DTU VI: light blue). A) Number of reads in each genome. B) Mapping rate of reads to *T. cruzi* assembly.

A

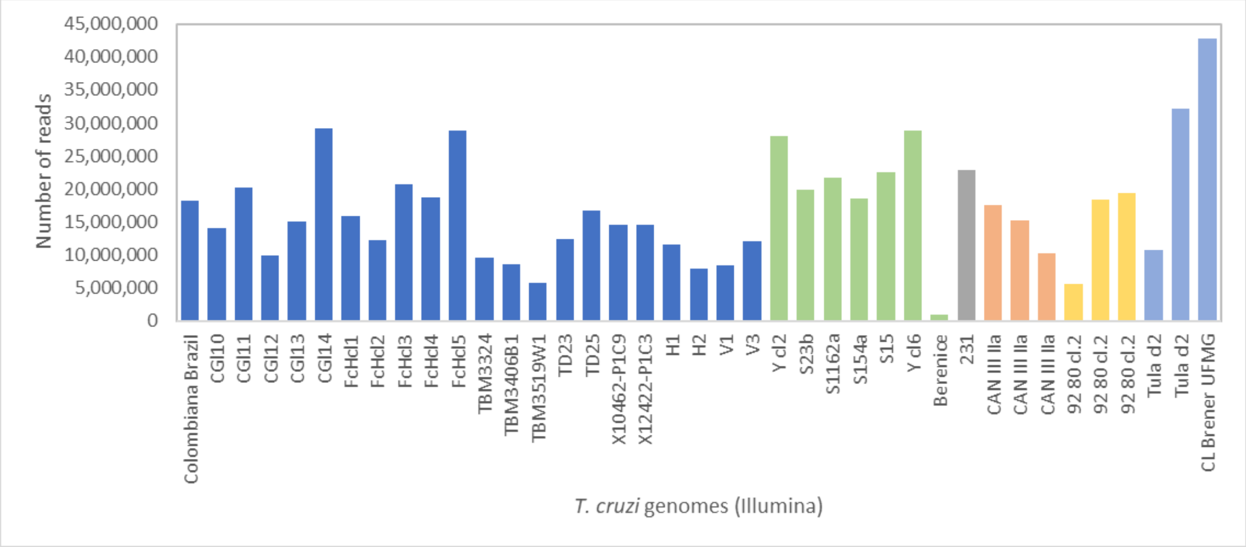

B

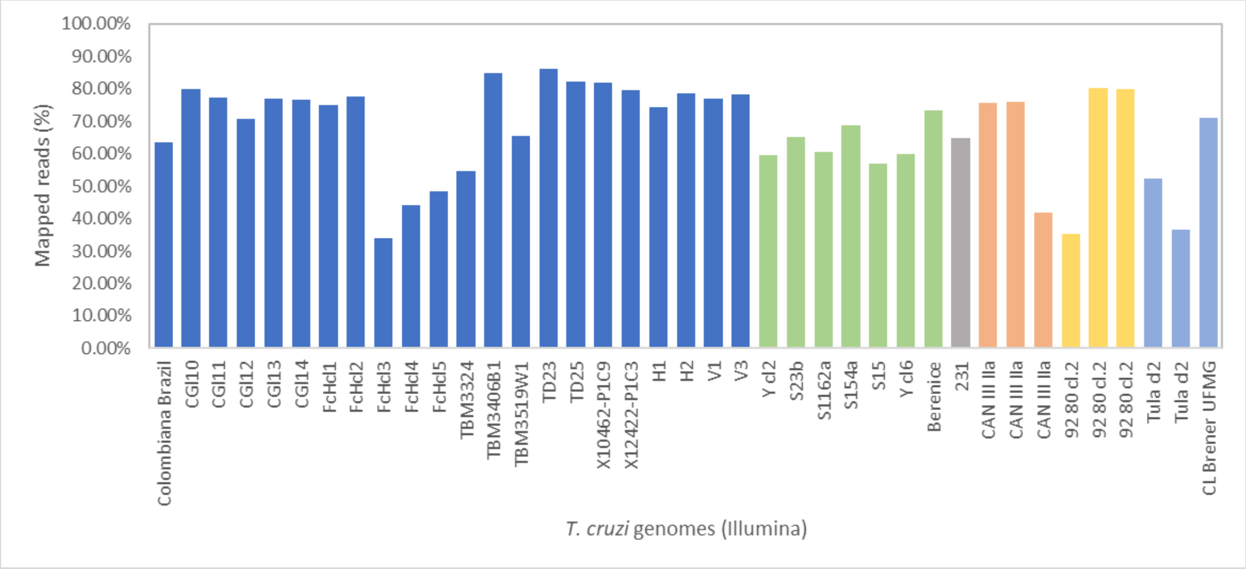

Supplementary Figure 9. Geographical location of the capture site of the reservoir *Didelphis marsupialis* for isolation by hemoculture of *T. cruzi*.

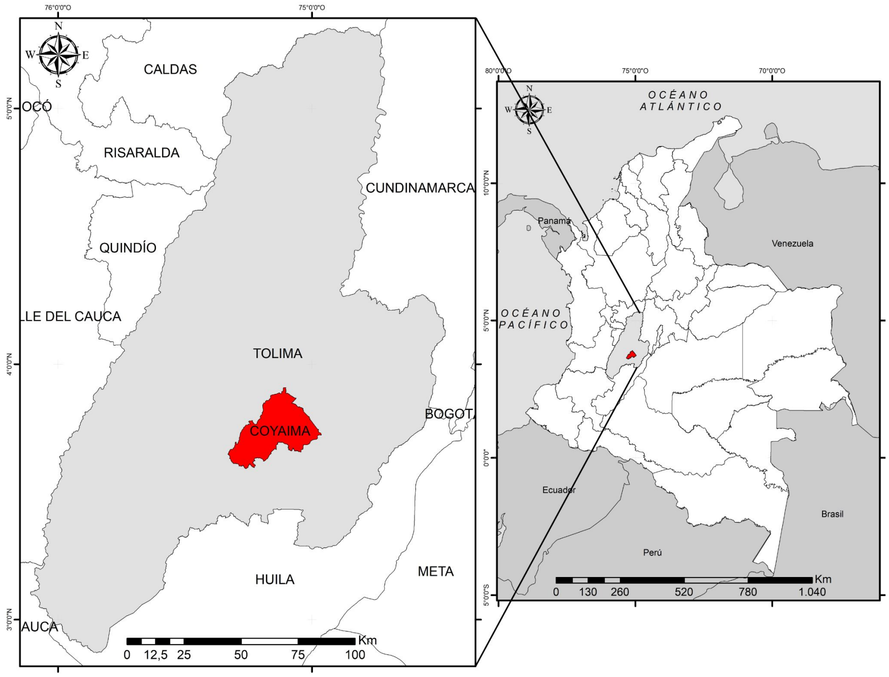

Supplement: Supplementary file 4 — Supplementary Information 4. [file 41598_2024_52449_MOESM4_ESM.pdf]
